# A Phylogenomic Perspective on Evolution and Discordance in the Alpine-Arctic Plant Clade *Micranthes* (Saxifragaceae)

**DOI:** 10.3389/fpls.2019.01773

**Published:** 2020-02-07

**Authors:** Rebecca L. Stubbs, Ryan A. Folk, Chun-Lei Xiang, Shichao Chen, Douglas E. Soltis, Nico Cellinese

**Affiliations:** ^1^Department of Systematic and Evolutionary Botany, University of Zürich, Zürich, Switzerland; ^2^Department of Biological Sciences, Mississippi State University, Mississippi State, MS, United States; ^3^Key Laboratory for Plant Diversity and Biogeography of East Asia, Kunming Institute of Botany, Chinese Academy of Sciences, Kunming, China; ^4^College of Life Sciences and Technology, Tongji University, Shanghai, China; ^5^Department of Biology, University of Florida, Gainesville, FL, United States; ^6^Florida Museum of Natural History, University of Florida, Gainesville, FL, United States; ^7^Genetics Institute, University of Florida, Gainesville, FL, United States; ^8^Biodiversity Institute, University of Florida, Gainesville, FL, United States

**Keywords:** arctic, alpine, diversification, coalescent, gene tree conflict, phylogenomics, incongruence, Saxifragaceae

## Abstract

The increased availability of large phylogenomic datasets is often accompanied by difficulties in disentangling and harnessing the data. These difficulties may be enhanced for species resulting from reticulate evolution and/or rapid radiations producing large-scale discordance. As a result, there is a need for methods to investigate discordance, and in turn, use this conflict to inform and aid in downstream analyses. Therefore, we drew upon multiple analytical tools to investigate the evolution of *Micranthes* (Saxifragaceae), a clade of primarily arctic-alpine herbs impacted by reticulate and rapid radiations. To elucidate the evolution of *Micranthes* we sought near-complete taxon sampling with multiple accessions per species and assembled extensive nuclear (518 putatively single copy loci) and plastid (95 loci) datasets. In addition to a robust phylogeny for *Micranthes*, this research shows that genetic discordance presents a valuable opportunity to develop hypotheses about its underlying causes, such as hybridization, polyploidization, and range shifts. Specifically, we present a multi-step approach that incorporates multiple checks points for paralogy, including reciprocally blasting targeted genes against transcriptomes, running paralogy checks during the assembly step, and grouping genes into gene families to look for duplications. We demonstrate that a thorough assessment of discordance can be a source of evidence for evolutionary processes that were not adequately captured by a bifurcating tree model, and helped to clarify processes that have structured the evolution of *Micranthes*.

## Introduction

The ability to sample hundreds of loci initially inspired optimism about recovering robust phylogenetic inferences despite discordance (Gee, 2003; Rokas et al., 2003). This optimism has been tempered by the growing finding that conflict is rampant throughout the tree of life at different taxonomic levels across phylogenomic datasets (e.g., Wickett et al., 2014; Crowl et al., 2017; Folk et al., 2018; Moore et al., 2018). Gene tree reconciliation methods seeking to deal with these problems have been developed, but commonly these methods and the studies using them treat discordance as a nuisance parameter and do not account for the possibility of the incongruence itself being of interest (Hahn and Nakhleh, 2016; Sayyari et al., 2018).

Here, we investigate the phylogeny and evolution of a complex clade of north temperate, alpine, and arctic flowering herbs for which inter- and intraspecific relationships have remained obscure. *Micranthes* Haw. (Saxifragaceae), a clade of ~80 species, occurs primarily in temperate, montane, and arctic habitats in the Northern Hemisphere (Brouillet and Elvander, 2009a). Previous phylogenetic investigations of *Micranthes* included only a few genes and/or limited sampling, and none have addressed the potential causes of phylogenetic conflict that have been documented (Lanning, 2009; Prieto et al., 2013; Tkach et al., 2015). One reason for this is that *Micranthes* is characterized by phylogenetic complexities variously attributed to autopolyploidy, allopolyploidy, aneuploidy, and hybridization. The hypothesized role of hybridization and polyploidy has been supported by the success of generating artificial hybrids and documentation of a wide range of chromosome numbers, both within and among species (Perkins, 1978; Elvander, 1984). Of the 34 species of *Micranthes* that have reported chromosome counts, the range spans from 2*n* = 10 to 120 with the most common counts being 2*n* = 20 and 38 (Rice et al., 2015). Extensive intraspecific variation also exists, e.g., *Micranthes occidentalis* has reported chromosome counts of 2*n* = 20, 38, 40, 56, 58 (Perkins, 1978; Brouillet and Elvander, 2009a). This substantial variation in chromosome numbers is unusual, not only within Saxifragaceae, but also angiosperms (Webb and Gornall, 1989; Soltis, 2007; Brouillet and Elvander, 2009b; Weiss-Schneeweiss and Schneeweiss, 2013).

We assembled and analyzed a phylogenomic dataset developed through target-enrichment and investigated these data with multiple complementary methods to assess robustness. We employed a gamut of methods to explore gene tree discordance, and we used this discordance to inform hypotheses regarding the evolution of *Micranthes*. Further, we assessed paralogous genes and analyzed species-tree *vs.* gene-tree conflicts. We then used the resulting best-supported phylogenetic hypothesis to compare dating methods for phylogenomic datasets. These analyses were used to address two main questions: 1) are different evolutionary narratives obtained when examining and accounting for incongruence? and 2) how can gene conflict be used to inform phylogenetic inference? Notably, in addition to addressing these questions we demonstrate that datasets with considerable phylogenetic conflict are not inherently intractable for subsequent analyses. This research illustrates methods for investigating conflicting signals in phylogenomic data, and provides refined inferences of phylogenetic and biogeographic relationships for *Micranthes*.

## Materials and Methods

### Taxon Sampling and Target Enrichment

Specimens were collected from natural populations spanning the Northern Hemisphere over three successive field seasons (2014–2016). Additional samples, primarily for outgroup taxa, were obtained from herbarium specimens and other field collections preserved in silica (**Table S1**). In all, our dataset comprised 161 samples, including 27 outgroups and 68 *Micranthes* (**Table S1**).

DNA extraction, probe design, target-capture, and dating analysis followed Stubbs et al. (2018), and are summarized briefly here. We used a target-capture approach for enrichment of pre-selected genomic regions optimized to provide resolution at multiple phylogenetic scales. To obtain transcriptomes fresh leaf tissue for six species of *Micranthes* species (*Micranthes petiolaris, Micranthes caroliniana, Micranthes careyana, Micranthes micranthidifolia, Micranthes oregana*, and *Micranthes ferruginea)* was collected from natural populations, placed in liquid nitrogen, and stored at −80°C for subsequent RNA extraction. RNA extractions were performed per option 2 of the protocol by Jordon-Thaden et al. (2015) with the addition of 20% sarkosyl. RNA library preparation and sequencing were performed by BGI (Shenzhen, China). Reads were filtered by quality-score prior to assembly, which was performed using SOAPdenovo-trans v1.03 (Xie et al., 2014) for the transcript (-K 35; -M 1, -F) assembly following Wickett et al. (2014).

Using the program MarkerMiner v1.0 (Chamala et al., 2015) the transcriptomes were input against reference databases composed of annotated single-copy genes in *Arabidopsis thaliana*. We used read-mapping in Geneious v9.0.2 (Kearse et al., 2012) to check for paralogous genes. If there were multiple overlapping hits from one transcriptome, this was considered a potential paralog and the gene was removed.

Probes universal to the clade Saxifragales were designed using parallel methods (Folk et al., 2019); sequence divergence was accounted for using up to 11 orthologs per gene from the 1KP project transcriptomes based on 11 species (Matasci et al., 2014). The order-level probes were multiplexed with the *Micranthes-*specific probe set, and in total, the final markers selected for probe design included 518 putatively single-copy nuclear genes. This gene set was used to design a myBaits custom bait library (formerly Mycroarray; Ann Arbor, Michigan, USA), with 120-mer biotinylated baits, an overlap of 60 base pairs (bp), and 3x tiling.

We selected taxa to supplement the 27 ingroup accessions from our previous analysis (Stubbs et al., 2018) with the aim of having a substantial representation of every clade of *Micranthes* based on the most recent treatment of the group (Tkach et al., 2015). Total genomic DNA was extracted from silica-dried and herbarium specimen leaf material, following a modified cetyl trimethylammonium bromide (CTAB) extraction protocol (Doyle and Doyle, 1987). Double-barcoded Illumina libraries were built by RAPiD Genomics (Gainesville, Florida), with a size selection step aiming for >200 bp. Target capture reactions were performed with either a custom myBaits kit either in-house following the v3.1 manual or by RAPiD Genomics with libraries pooled 8-plex. The post-capture libraries were pooled and sequenced on six lanes of an Illumina HiSeq 3000 with 150 bp paired-end reads.

### Phylogenetic Analysis

Assembly of the nuclear reads was performed using HybPiper v1.2 (Johnson et al., 2016), a collection of Python scripts that employs the original gene sequences used for probe design to assemble sequences for each target locus. Post-processing scripts were run in HybPiper to retrieve both a summary of target enrichment and gene recovery efficiency and a heat map visualizing the gene recovery efficiency per sample. Chloroplast genes were assembled from off-target reads following the same methods as the nuclear dataset, but using as a reference the complete plastome of *Heuchera parviflora* var. *saurensis* (Folk et al., 2015), with the addition of intergenic regions identified using DOGMA (Wyman et al., 2004). All genes and spacer regions were manually reviewed for length and quality, and genes and spacers >200 bp were kept. In total, we retrieved 95 chloroplast coding sequences and intergenic regions.

Additionally, we ran the post-processing script “paralog_investigator.py” on the nuclear dataset to extract coding sequences from alternative contigs flagged by HybPiper (available from https://github.com/mossmatters/HybPiper). This tool determines potential paralogs as follows: first, the initial retrieval script in HybPiper identifies all contigs matching each probe, then a single contig is selected based on size and similarity and the smaller contigs are flagged and removed, and finally, the post processing script retrieves all flagged contigs for subsequent use (Johnson et al., 2016). To assess if these additional contigs represented paralogs, homeologs, allelic variation, or contamination, the files generated by HybPiper containing all gene contigs (both retained and discarded) for all species were retrieved and run through a pipeline to create a phylogeny. This pipeline consisted of an alignment in MAFFT v7.215 (Katoh and Standley, 2013) with default parameters and a tree reconstruction using FastTree 2 (Price et al., 2010) specifying the GTR+CAT model. All resulting gene trees were manually examined, and any genes that included multiple contigs for multiple species were removed from downstream analyses. In all, 37 genes were discarded at this step, resulting in a final collection of 481 putative single copy nuclear genes for downstream analyses.

Methods of alignment and analyses followed Stubbs et al. (2018) and are reviewed here. The 481 single-copy loci and plastid genes were individually aligned with MAFFT. Alignment statistics were calculated by AMAS (Borowiec, 2016). In both datasets, genes were concatenated and partitioned, and a maximum likelihood (ML) analysis was performed using RAxML v8 (Stamatakis, 2014). Gene trees were inferred for each of the individual nuclear gene alignments.

A coalescent species tree was inferred from the 481 best ML nuclear gene trees from RAxML and 1,000 bootstraps (BS), using ASTRAL-II v5.0.3 (Mirarab and Warnow, 2015). Branches with less than 10% BS were collapsed in the gene trees using Newick utilities (Junier and Zdobnov, 2010). We examined coalescent branch length estimated in ASTRAL-II to quantify incomplete lineage sorting (ILS) expectations throughout the tree. Although this metric is conservative since all gene incongruence is assumed to be due to ILS, it is useful as a direct measure of the amount of discordance in the gene trees. Support of the ASTRAL-II species tree was quantified using the local posterior probability (LPP) of a branch as a function of its normalized quartet support (Sayyari and Mirarab, 2016). We first ran ASTRAL-II with all accessions, and from that resulting tree we collapsed all species that were recovered as monophyletic. Additionally, to use the ASTRAL-II results for downstream analyses requiring branch lengths, we used RAxML to optimize branch lengths for this topology by constraining RAxML to the ASTRAL-II topology. We define the support on the species trees from here on as follows: strong support will correspond to either BS ≥90 or LPP ≥0.9 and poor support will be any node with BS <70 or LPP <0.9. All trees were visualized with FigTree v1.4.2 (Rambaut, 2009) and Interactive Tree of Life (iTOL) (Letunic and Bork, 2016).

**Figure 1:**
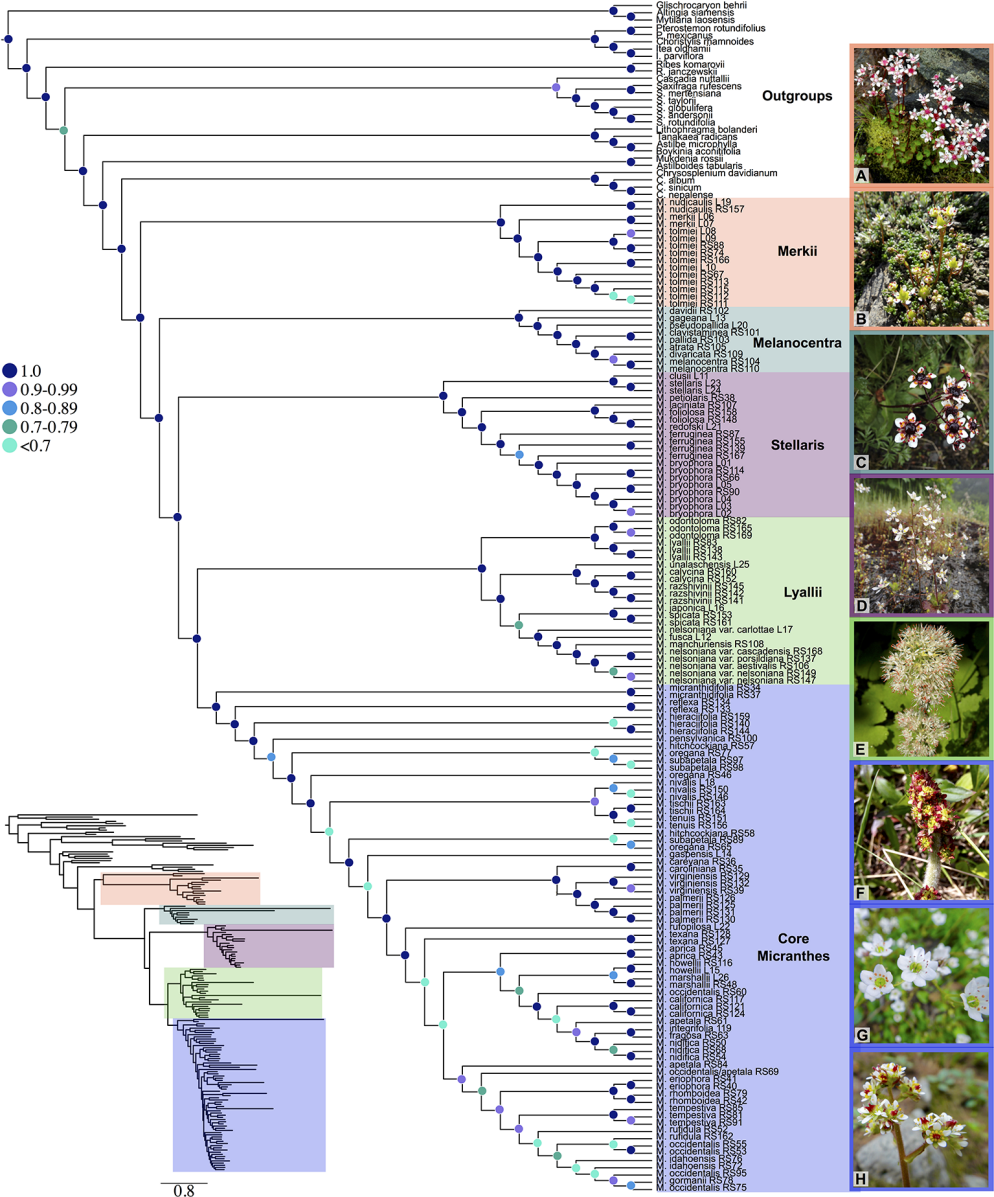
Species tree generated using ASTRAL-II and 481 low-copy nuclear genes. Nodes are colored corresponding to local posterior probability. Additionally, clades are colored to show boundaries of each clade. Inset shows branch lengths generated in RAxML for the ASTRAL-II topology. **(A)** *Micranthes nudicaulis*, **(B)** *Micranthes tolmiei*, **(C)** *Micranthes melanocentra*, **(D)** *Micranthes ferruginea*, **(E)** *Micranthes manchuriensis*, **(F)** *Micranthes hieraciifolia*, **(G)** *Micranthes californica*, **(H)** *Micranthes tempestiva*. All photos by RLS.

Numerous sections and series have been designated within *Micranthes* (Haworth, 1821; Don, 1822; Johnson, 1934; Gornall, 1987; Soltis et al., 1996; Tkach et al., 2015; Gornall, 2016). For ease of discussion we will be referring to the five clades that are maximally supported (LPP = 1.0) in our results. These clades are *Merkii*, *Melanocentra, Stellaris, Lyallii*, and the core *Micranthes* (**Figure 1**).

Conflict between gene trees and the species tree was assessed using PhyParts v0.0.1 (Smith et al., 2015). For input into PhyParts, all gene trees were rooted using Phyx (Brown et al., 2017) and subsequently outgroups were removed. To minimize the effect of gene tree estimation error, we applied a bootstrap filter where edges with bootstrap values lower than 70% were ignored for congruence calculations. We ran PhyParts on both the dataset with all accessions and a reduced dataset where the gene trees were pruned to one species per accession. When two or more accessions were available per species, the accession with the most nuclear exon sequence data was selected. To visualize the results we used scripts developed by Matt Johnson (https://github.com/mossmatters/MJPythonNotebooks/blob/master/PhyParts_PieCharts.ipynb). We further explored gene conflict using the gene- and site-concordance factors in IQ-TREE (Nguyen et al., 2015; Minh et al., 2018); gCF is the percentage of gene trees containing a specific branch in the species tree, while sCF is the percentage of alignment sites supporting that branch. For input into IQ-TREE we used the ASTRAL-II species trees and the RAxML gene trees and 500 quartets were randomly sampled per branch. Finally, incongruence between the chloroplast and nuclear genome was visualized using the “tanglegram” function in DensiTree.

### Gene Duplication

We clustered gene families using OrthoFinder (Emms and Kelly, 2015) under the default parameters, with the exception of providing our rooted ASTRAL-II species tree for reconciling the gene trees. The input files for OrthoFinder were all exons plus potential paralogs as retrieved by HybPiper. First, we reduced our dataset to just ingroup taxa, removed two taxa with low coverage (*Micranthes pseudopallida* and *Micranthes japonica*), and used only a single accession per species (as above). As we were interested in paralogs, we included all contigs for each of the original 518 genes. The summary statistics output by OrthoFinder included a list of nodes that were recovered as having a duplication event, the genes included in that duplication, and the support of the duplication for that node. To ensure that short contigs were not misinterpreted as gene duplications and that multiple orthogroups were not constructed from the same gene, we carefully examined the output from OrthoFinder. We found no examples of either scenario.

### Molecular Dating

We employed multiple approaches for divergence-time inference. For all approaches we used the same fossil constraints. The best well-documented fossils to use for *Micranthes* are in closely related families outside Saxifragaceae, including fossilized leaves of *Ribes webbii* in Grossulariaceae (Hermsen, 2005), *Itea* fossil pollen in Iteaceae (Hermsen, 2013), and fossils of *Divisestylus* in the Saxifragaceae alliance (Hermsen et al., 2006; Hermsen, 2013). Fossils were applied following the placements suggested in previous dating analyses of Saxifragaceae (Ebersbach et al., 2016; Stubbs et al., 2018; Folk et al., 2019). Briefly, fossil constraints were as follows: constraining the stem *Itea* + *Choristylis* + *Pterostemon* at 49 million years ago (Mya); placing the most recent common ancestor (crown) age of *Ribes* at 14.5 Mya, and the crown of the Saxifragaceae alliance (all taxa excluding *Mytillaria*, *Glischrocaryon, Altingia*) at 89 Mya.

Dating analyses were conducted using Bayesian methods. Given the computational resources needed for these methods, it was necessary to reduce the scale of the datasets *via* “gene shopping” methods (Hedges et al., 1996; Kumar and Hedges, 1998); such methods seek to identify a reduced set of genes with the best information relevant to time calibration. One method followed Johns et al. (2018) and employed HashRF (Sul and Williams, 2008) to calculate the Robinson-Foulds (RF) distance (Robinson and Foulds, 1981) between the gene trees and the ASTRAL-II-constrained RAxML topology; those with the least RF distance have greater concordant phylogenetic signal. The 25 loci closest in RF distance to the species tree were selected. Secondly, we implemented an approach developed by Smith et al. (2018). We used the SortaDate package (https://github.com/FePhyFoFum/sortadate) to filter the 25 “best” loci. This package determines which gene trees are clock-like, have the least topological conflict with the species tree, and have informative branch lengths. Both datasets were concatenated for use in BEAST v1.8.2 (Drummond et al., 2012).

BEAST analyses were run under a relaxed uncorrelated lognormal model for 10^8^ generations, logging parameters every 2,000 generations, and assuming a coalescent process. The prior distribution for all fossil calibration points was lognormal with the aforementioned dates used as the median to determine the offset value. The molecular clock was set as relaxed lognormal. To speed up the analysis, five strongly supported recovered clades (see *Results*) were constrained, and five identical runs were carried out simultaneously. To summarize the results, all tree sets were combined using LogCombiner v1.8.2 after removing the burn-in (10% of trees). Summary statistics were calculated using TreeAnnotator v1.8.2.

## Results

### Target Enrichment Phylogenetic Analysis

In total, we captured up to 518 nuclear loci across all 164 accessions (**Figure S1**). All sequence data have been deposited at the Sequence Read Archive (BioProject: PRJNA587870). With the exception of the OrthoFinder results below, all *Results* and *Discussion* hereafter refer to the reduced low-copy 481-loci dataset. For gene recovery, the average locus length for *Micranthes* was 865 bp (range: 113–2,191 bp) and 49.8% (range: 7.5–75.7%) of reads were on target (**Table S2**). For ingroup taxa, the total alignment for the 481-loci matrix consisted of 665,502 base pairs, with 309,742 parsimony-informative sites, and had 22% missing data (**Table S3**). All analyses indicated that *Micranthes* consists of five lineages approximating traditionally recognized sections (**Figure 1**). We recovered monophyly for most species for which there were multiple samples, but there were a few exceptions from multiple sections, including *Micranthes nelsoniana*, *Micranthes hitchcockiana*, *Micranthes subapetala*, *M. oregana*, *Micranthes razshivinii*, and *Micranthes calycina*. Most polyphyletic species across all analyses were within the core *Micranthes* clade, which also included the highest number of low support values.

**Figure 2:**
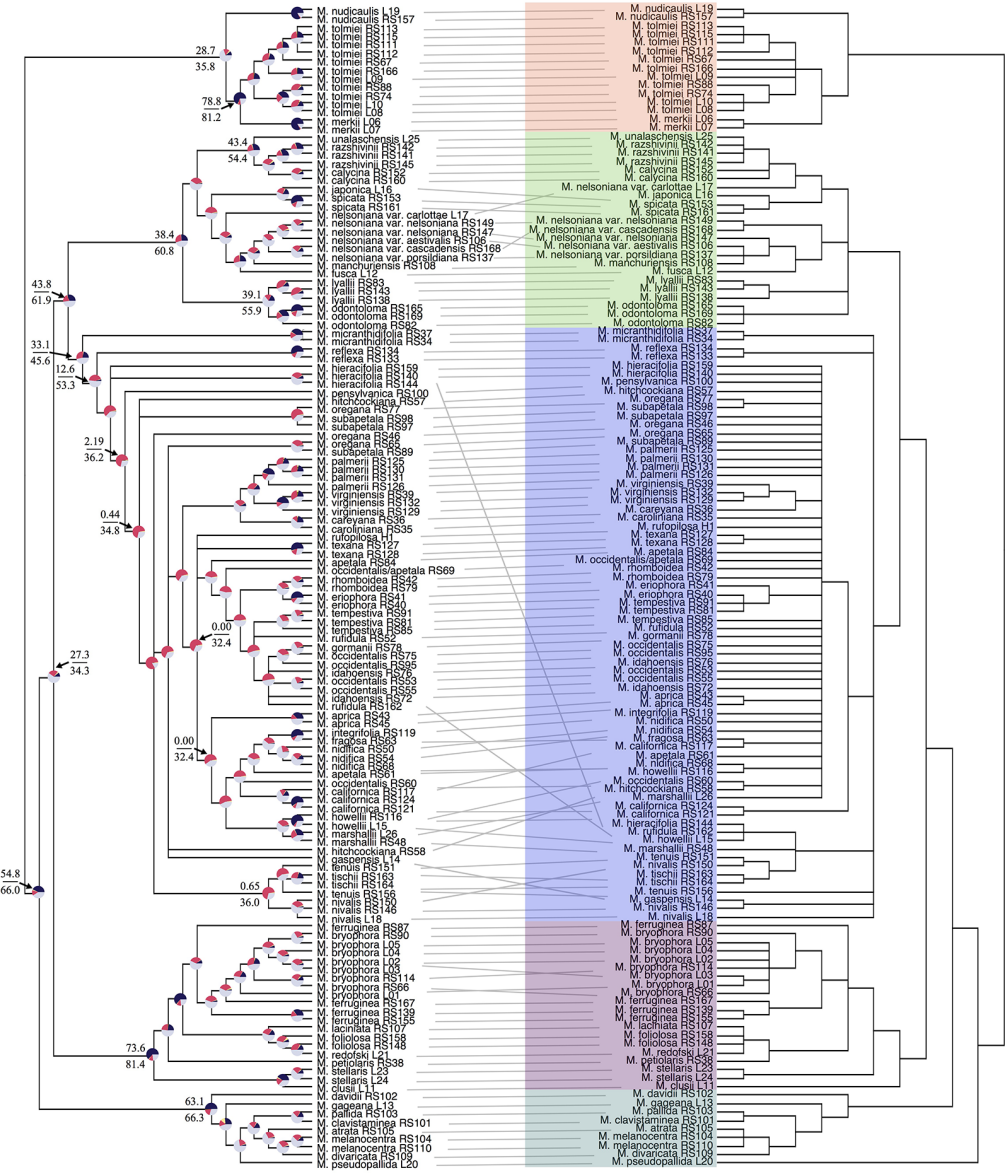
Tanglegram comparing ASTRAL-II nuclear species tree (left) and the RAxML plastid topology (right). Branches with less than 0.7 local posterior probability (LPP) or 70% bootstraps (BS) are collapsed. Clades are colored corresponding to **Figure 1**. Pie charts at nodes on ASTRAL-II tree show gene tree conflict evaluations at each node as the following: proportion of gene trees in concordance (blue), in conflict (pink), agreeing with the dominant alternative topology (yellow), and unsupported with less than 70% BS (gray). Select nodes also include the gene (above) and site (below) concordance factors. All concordance factors shown in **Supplementary Figure S6**.

Our off-target reads recovered substantial coverage of the plastome. In total, our plastid dataset consisted of 50,009 base pairs, 44% missing data, and 21% parsimony-informative for ingroup taxa (**Table S3**). Support in the plastid phylogeny was generally strong at deeper nodes and in most sections, but in the core *Micranthes* clade, there was poor support throughout (**Figure 2**).

The ASTRAL-II topology (**Figure 1**, **Figure S2**) was largely congruent with the RAxML concatenated dataset (**Figure S3**), and the main clades were maximally supported. Approximately 85% of branches in the *Micranthes* ASTRAL-II species tree with one accession per species (**Figure S2**) were less than one coalescent unit in length, consistent with significant discord due to ILS.

Conflict between the ASTRAL-II nuclear topology and plastid phylogenetic trees is shown *via* a tanglegram in **Figure 2**, where nodes with <70% BS and <0.7 LPP are collapsed. Both analyses recovered maximal support for the *Micranthes* clade as a whole and for five distinct clades. The *Merkii*, *Stellaris*, and *Melanocentra* clades did not have any hard interspecific incongruence (> 70% BS, > 0.7 LPP) between the nuclear and chloroplast trees. In the *Lyallii* clade relationships were generally congruent with the exception of relationships in *M. nelsoniana* and the placement of *Micranthes unalaschensis* as either sister to *M. razshivinii* + *M. calycina* (nuclear) or nested within *M. razshivinii* (plastid). There were multiple instances of incongruence throughout the core *Micranthes* clade, and the relationships within this clade were recovered with the lowest support in both the nuclear and plastid trees.

**Figure 3:**
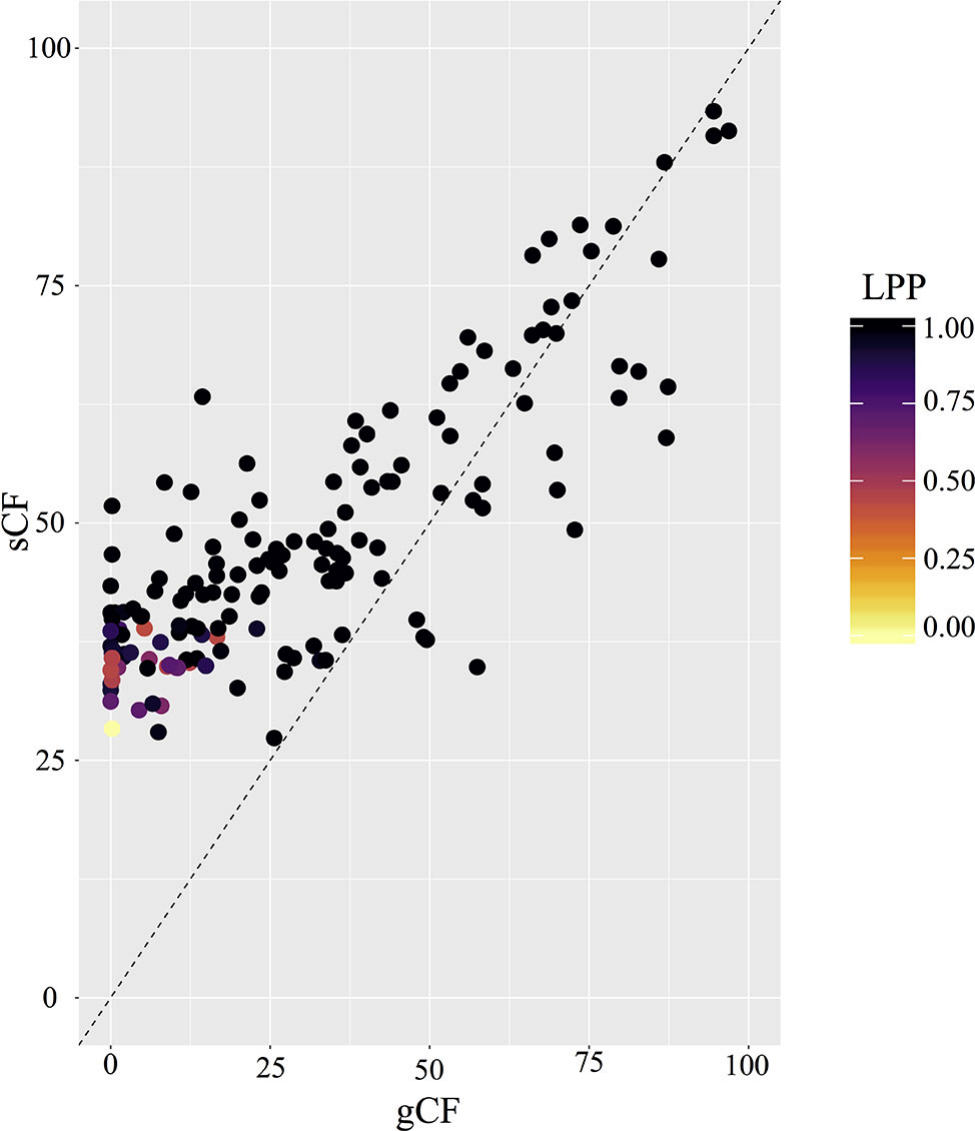
Comparison of gCF (gene concordance factor) and sCF (site concordance factor) by local posterior probability at each node in species tree.

PhyParts recovered many instances of discordance in the nuclear gene trees—more than two-thirds of nodes in the ASTRAL-II tree were shown to have more (> 60%) gene trees in disagreement with that node than in agreement (**Figure 2**, **Figures S4** and **S5**). IQ-TREE further supported the high amount of discordance in our dataset. For many of the branches within the *Micranthes* phylogeny, the two concordance factors—gene concordance factors (gCF) and site concordance factors (sCF)—are similarly low regardless of high LPP. The nodes with the lowest LPP also had the lowest gCF and sCF, yet even with relatively low gCF and sCF we still recovered maximal support for many nodes (**Figure 3**).

### Duplications in Genes, Gene Families, and Genomes

In the original 518-nuclear gene dataset, both HybPiper and OrthoFinder recovered many potential paralogs. HybPiper identified potential paralogs in 110 of 138 ingroup accessions. Notably, the five accessions that had the most genes with detected paralogs based on HybPiper—*M. hitchcockiana* RS57, *M. subapetala* RS98, *Micranthes pensylvanica* RS100, *M. subapetala* RS97, and *M. oregana* RS77—had either low bootstrap support or incongruent placements in all species trees. Of those five species, all but *M. pensylvanica* had more than one accession in our analyses, and the other accession(s) for each species did not have many paralogs, nor were they recovered as monophyletic. This lack of monophyly, however, could be an artifact of lower sequence coverage for those other accessions (**Table S2**).

OrthoFinder, which in contrast to HybPiper investigates deep paralogy patterns of gene families and not just individual taxon paralogs, also recovered putative gene duplication events. OrthoFinder recovered 301 gene family duplications. At terminal nodes, the most duplications were recovered for *Micranthes atrata, Micranthes pallida*, and *Micranthes melanocentra*. The most strongly supported duplications (40% or more of the daughter taxa at that node) were shared across 12 nodes, and the most recent common ancestor (MRCA) for the *Stellaris* clade had the most duplications.

### Molecular Dating

The two gene shopping methods selected six of the same loci, but the other 19 loci varied between the two datasets. The concatenated SortaDate dataset (**Figure S7**) was a total of 42,340 bp, while the RF dataset (**Figure S8**) was a total of 36,778 bp. These methods recovered slightly different topologies compared to the ASTRAL-II topology. The RF phylogeny differed from the ASTRAL-II species tree and the SortaDate phylogeny in the placement of the *Stellaris* clade. There were additional differences between all three topologies at the terminals, and therefore, our *Results* and *Discussion* will be focused on deeper nodes. In spite of these differences, the dates recovered by the gene shopping methods were similar to each other (**Table 1**). For instance, the crown age of *Micranthes* was estimated to be 52.8 Ma (95% HPD = 40.2–69.9 Ma) in the RF dataset, and it was dated slightly older with the SortaDate genes at 58.3 Ma (95% HPD = 38.9–79.7 Ma). The crown age of the core *Micranthes* was dated to 18.0 Ma (95% HPD = 11.6–24.1 Ma) in the RF dataset, but with the SortaDate genes the crown clade was dated slightly younger to 17.3 Ma (95% HPD = 7.8–24.4 Ma). Due to the similarities in divergence estimates between the two gene shopping methods, and that there were more topological differences between the RF dataset and our ASTRAL-II species tree, we used the dated phylogeny generated by the 25 best genes selected by SortaDate for the discussion.

**Table 1:** Divergence time estimates for major clades within *Micranthes* for two gene shopping methods.

Node	SortaDate		Robinson-Foulds Distance	
	Mean	95% HPD	Mean	95% HPD
*Micranthes* group crown	58.3	38.9–79.8	52.8	40.2–69.9
*Merkii* group crown	46.9	27.4–67.7	27.5	14.0–41.0
*Melanocentra group crown*	33.4	16.7–60.7	20.3	7.3–31.7
*Stellaris group crown*	14.0	7.4–21.4	18.0	12.0–27.1
*Lyallii* group crown	14.5	7.4–28.0	15.4	7.3–24.0
Core *Micranthes* group crown	17.3	7.8–24.4	18.0	11.6–24.1
Core *Micranthes* + *Lyallii* group crown	25.1	15.2–39.4	24.8	16.9–32.6

HPD, highest posterior density.

## Discussion

An important initial goal of this study was to generate a phylogenetic hypothesis for *Micranthes*; our analyses provided robust support for the major relationships within this clade (**Figure 1**). Strong support for most relationships throughout *Micranthes* was recovered, although the core *Micranthes* clade consistently had the lowest support. Because one of the goals of our research was to investigate genetic conflict, we used multiple tools to highlight discordance in our dataset. These areas of incongruence do not necessarily hamper the ability to generate testable hypotheses through downstream analyses. Hereafter, we will focus only on a few selected key conflicts that are exemplary of our methods for examining discordance.

The ancestral node for *Micranthes* was reconstructed with high support in all of the analyses, and both PhyParts and IQ-Tree concordance factors suggest that most loci support the relationships at the nodes. The BEAST dating analysis based on the SortaDate genes (**Table 1**, **Figure S7**) showed that the ancestor of *Micranthes* started diversifying at 58.8 Ma (95% HPD = 38.9–79.8 Ma). This date is near the Cretaceous–Paleogene (K–Pg) boundary (65 Ma), and it is widely accepted that a major perturbation, such as a catastrophic mass extinction event, would clear ecological space, reducing competition, and allow the adaptive radiation of formerly restricted groups (Wing and Sues, 1992). Consistent with this theory, during the late Paleocene and early Eocene the Earth was much warmer than at present, but the high Arctic had a climate similar to present day Idaho, Montana, and Colorado. Hence, the suitable habitat for this cold-adapted clade would have been primarily polar in a largely tropical-subtropical globe (Harrington et al., 2012).

The *Melanocentra* and *Stellaris* clades have maximal support at all but three nodes (**Figure 1**). There is limited gene conflict at the base of these clades, but an increase of discordance at shallower nodes. This conflict could be attributed to paralogs. In the *Melanocentra* clade, *M. atrata*, *M. pallida*, and *M. melanocentra* had the highest number of gene family duplications, and in conjunction, many of the branches subtending these species have a gCF that is notably lower than the sCF (**Figure 2**). Generally, when the gCF values are affected by processes other than discordance, the gCF values will be lower than sCF values (Lanfear, 2018). Therefore, the high posterior probabilities recovered for this clade suggest that the species coalescent method was able to reconcile the gene trees and species trees, but some of the loci in the analysis are not constrained to bifurcating speciation events.

The *Stellaris* clade contained the most gene family duplications with high support. Although four duplicated gene families represent a small percentage of all gene families recovered, this is likely a significant underestimate of the background genome duplication rate, given our *a priori* filtering for genes generally maintained as single-copy (De Smet et al., 2013). Hence, a possible explanation for these gene duplications is that they resulted from an ancestral polyploidy event. There are multiple lines of evidence for this theory. For one, this clade is associated with the production of bulbils in place of flower buds, a form of asexual reproduction not seen in any other *Micranthes*. This is notable because asexual reproduction and whole genome duplication are correlated, with polyploids displaying elevated rates of asexual propagation compared to diploid relatives (Baduel et al., 2018). Additionally, four species from this clade have chromosome numbers reported in the literature: *M. ferruginea* (2*n* = 20, 38*), Micranthes foliolosa* (2*n* = 40, 48, 56, 64*)*, *Micranthes clusii* (2*n* = 28), and *Micranthes stellaris* (2*n* = 28; Brouillet and Elvander, 2009a; Rice et al., 2015). Taken together, this combination of results suggests that the *Stellaris* clade is an ideal group for further investigation into a series of putative whole genome duplications.

A well-supported example of putative hybrid speciation is recovered within the *Lyallii* clade for three species with overlapping distributions in Alaska. In the ASTRAL-II tree, *Micranthes unalaschensis* is recovered as sister to both *M. calycina* + *M. razshivinii* (**Figure 1**), while in the plastid tree *M. unalaschensis* is placed in a clade with just *M. razshivinii* (**Figure 2**). In contrast, in the RAxML tree (**Figure S3**), *M. unalaschensis* is recovered in a clade with just *M. calycina*. Additionally, in the ASTRAL-II tree the coalescent length for the branch subtending this clade was near one, suggestive of low ILS especially under organelle inheritance (Folk et al., 2016). This is also indicative of hybridization and chloroplast capture rather than ILS for explaining the chloroplast topology. Further, it has been suggested by previous studies that *M. calycina* may hybridize with both *M. unalaschensis* and *M. razshivinii* due to the discovery of morphologically intermediate plants in Alaska (McGregor, 2008; Brouillet and Elvander, 2009a). This evidence for hybridization and overlapping distributions, in combination with our molecular analysis, suggests that *M. unalaschensis* could be the result of a hybrid speciation event, with plastid genes consistent with a relationship with *M. razshivinii*, and nuclear genes suggesting a relationship with *M. calycina*.

The core *Micranthes* clade is recovered in all of our analyses as having a high level of discordance throughout the clade. PhyParts recovered many gene trees being in disagreement with both each other and the species tree (**Figure 2**, **Figures S4** and **S5**), and almost never recovered a single dominant alternative topology (yellow pie slice). This discordance could be the result of ILS, hybridization, and/or gene duplication (see below). Furthermore, in the ASTRAL-II tree (**Figure S2**; measured in coalescent units) and the dated phylogeny (**Figure S7**; measured in millions of years), the core *Micranthes* clade had very short branch lengths, also characteristic of rapid radiations (Folk et al., 2015; Hutter et al., 2017; Mitchell et al., 2017). The branch subtending this clade was of length ~1 suggesting low levels of discord between the core *Micranthes* and other clades.

We recovered multiple examples of strongly supported phylogenomic conflict in the core *Micranthes* (**Figures 1** and **2**). A notable area of conflict is the evolutionary history of *M. hitchcockiana* and *M. subapetala*. For one, both *M. hitchcockiana* and *M. subapetala* have chromosome counts representative of tetraploids of 2*n* = 76 (Perkins, 1978; Elvander, 1984; Brouillet and Elvander, 2009a). Additionally, four decades ago Perkins (1978) and Elvander (1984) suggested that *M. hitchcockiana* may have arisen as the result of genome duplication following hybridization between *M. oregana* and a member of the *M. occidentalis* complex (including *M. occidentalis*, *Micranthes rufidula, Micranthes idahoensis*, and *Micranthes gormanii*), and that *M. subapetala* was an autopolyploid with *M. oregana* as its progenitor. This hypothesis was based on exceptional morphological, ecological, and artificial hybridization studies, and we can now further test this hypothesis with molecular data.

In our analyses, *M. hitchcockiana*, *M. oregana*, and the *M. occidentalis* complex are consistently supported as being polyphyletic in both plastid and nuclear phylogenies. Specifically, both individuals of *M. hitchcockiana* (collected from different populations) are recovered in separate clades with different accessions of *M. oregana* and *M. subapetala*. These results, taken together with the detailed morphological, cytological, and field studies conducted by Perkins (1978) and Elvander (1984), do support the hypothesis that *M. subapetala* and *M. hitchcockiana* are the result of polyploid speciation. For *M. hitchcockiana*, our analyses, in combination with previous work, suggest this species is an allopolyploid and that the other progenitor is from the *M. occidentalis* complex, again aligning with the predictions from Elvander (1984) and Perkins (1978). The molecular analyses did not recover any signal for the other progenitor of *M. subapetala*. Therefore, at least four other possibilities exist: 1) the other progenitor is extinct; 2) the other progenitor is a cryptic species we did not sample; 3) *M. subapetala* is the result of a hybridization event followed by a backcross to *M. oregana*, thus, masking the signal of the other parent; or 4) *M. subapetala* is an autopolyploid as previously hypothesized. Our analyses suggest that due to these taxa being polyphyletic and having a divergence time of approximately 5 Ma (**Figure S7**), *M. hitchcockiana* and *M. subapetala* may have multiple origins of a polyploid taxa, a scenario believed to be prevalent in natural populations (Soltis and Soltis, 1999; Soltis et al., 2015). Further tests are needed to fully explore these hypotheses.

We used multiple lines of evidence to uncover diverse evolutionary dynamics in *Micranthes*, including hybridization, introgression, and polyploidization. *Micranthes* is a complex clade, representing a diverse radiation, and likely involving many cryptic species. Overall, our methods generated a resolved phylogeny of *Micranthes* across multiple taxonomic levels despite much underlying conflict. More work is needed on this group, particularly to understand chromosome, gene family, and ploidal evolution, but we were able to reconstruct multiple, well-supported instances of putative non-bifurcating evolution.

Due to the complicated evolutionary history of *Micranthes*, as inferred by our data and previous analyses, we suggest that designations of traditional species by morphology and/or shallow genetic sampling may be misleading. In fact, our results indicate that lower support values seen in shallow relationships do not reflect a lack of phylogenetic signal, but are likely the product of evolutionary processes not adequately captured by a bifurcating tree model. Teasing apart these varied phylogenetic histories required explicit assessments of orthology and detailed evaluations of discordance; this points to the importance of orthology assessment in phylogenomics and explicit consideration of conflicting evolutionary histories (Crowl et al., 2017; Folk et al., 2018). A multi-step approach, such as the one used here, incorporating four check points for paralogy—targeting putatively single-copy genes in our bait design, reciprocally blasting targeted genes against transcriptomes, running paralogy checks during the assembly step, and grouping genes into gene families to look for duplications—should result in more robust phylogenetic inference. Further, examining causes of discordance and low support values through multiple avenues can also inform evolutionary reconstructions. Subsequently, we recommend a similar approach for other challenging groups. Investigating conflicting phylogenetic signals provides the opportunity to unravel different evolutionary narratives, yielding new insights into speciation.

## Data Availability Statement

The datasets generated for this study can be found in the NIH Short Read Archive. BioProject: PRJNA587870.

## Author Contributions

RS, DS, and NC designed the research. RS, RF, and C-LX collected samples. C-LX and SC provided material and analyses. RS and RF conducted analyses and analyzed the results. RS, RF, DS, and NC wrote the manuscript.

## Funding

This work was funded by Torrey Botanical Society Graduate Student Research Fellowship, Arkansas Native Plant Society Delzie Demaree Research Grant, Cactus and Succulent Society of America Research Grant, The Explorers Club Exploration Fund–Mamont Scholars Program, Alaska Geographic Murie Science and Learning Center Science Education Grant, Arctic Institute of North America Grant-in-Aid, Washington Native Plant Society Research Grant, Botanical Society of America Graduate Student Research Award, National Science Foundation East Asia and Pacific Summer Institute Fellowship OISE 151227, Davis Graduate Fellowship in Botany, California Native Plant Society, Natalie Hopkins Award, Idaho Native Plant Society Education, Research and Inventory Grant Program, American Society of Plant Taxonomist Graduate Student Research Grant, Native Plant Society of Oregon Field Research Grant, Society of Systematic Biologist Graduate Student Award, John Paul Olowo Memorial Fund Research Grant (all to RS), National Science Foundation Grant DBI 1523667 (to RF), and National Science, Foundation Grant DBI 1458640 (to DS).

## Conflict of Interest

The authors declare that the research was conducted in the absence of any commercial or financial relationships that could be construed as a potential conflict of interest.

## Supplementary Material

Figure S1 Heatmap showing success of target capture. Darker colors represent higher capture success.

Figure S2 ASTRAL-II topology with single species accessions and outgroups removed. Branch lengths labeled in coalescent units.

Figure S3 RAxML analysis of concatenated nuclear dataset. Nodes are labeled with bootstrap support values.

Figure S4 PhyParts results depicting gene tree conflict with the ASTRAL-II species topology. Pie charts show gene tree conflict evaluations at each node as the following: proportion of gene trees in concordance (blue), in conflict (pink), agreeing with the dominant alternative topology (yellow), and unsupported with less than 70% BS (gray). The number above the branch is the number of gene trees that agree with the relationships at that node and the number below the branch is the number of gene trees that are in conflict with that node. Total number of trees is 481.

Figure S5 PhyParts results depicting gene tree conflict with the ASTRAL-II species topology where all but one accession per species were removed in the species tree and gene trees. Pie charts show gene tree conflict evaluations at each node as the following: proportion of gene trees in concordance (blue), in conflict (pink), agreeing with the dominant alternative topology (yellow), and unsupported with less than 70% BS (gray). The number above the branch is the number of gene trees that agree with the relationships at that node and the number below the branch is the number of gene trees that are in conflict with that node. Total number of tree is 478.

Figure S6 Tree showing posterior probability, gene concordance factor, and sequence concordance faster (in that order) for each branch. The gCF is the percentage of decisive gene trees containing that branch. The sCF is the percentage of alignment sites supporting a branch in the reference tree. Note that unlike gCF values, the sCF values sum to 100% because sCF values are calculated by comparing the three possible resolutions of quartet around a node.

Figure S7 BEAST dating analysis with SortaDate genes. Ages of nodes shown in millions of years and as the 95% HPD.

Figure S8 BEAST dating analysis with RF genes. Ages of nodes shown in millions of years and as the 95% HPD.
