# Supplementary material for: A Phylogenomic Perspective on Evolution and Discordance in the Alpine-Arctic Plant Clade Micranthes (Saxifragaceae)
Source: Front Plant Sci. 2020 Feb 7;10:1773. doi: 10.3389/fpls.2019.01773 (PMC7020907; doi:10.3389/fpls.2019.01773)
Supplement: Supplementary file 1 [file DataSheet_1.pdf]

Table S1. Specimens used in this study.

Taxa list	accession number (*Micranthes* only)	Voucher information (Herbarium)	collecting locality
Micranthes apetala (Piper) Small	RS61	R. L. Stubbs 61 (FLAS)	US: Washington
Micranthes apetala (Piper) Small	RS84	R. L. Stubbs 84 (FLAS)	US: Montana
Micranthes aprica (Greene) Small	RS43	R. L. Stubbs 43 (FLAS)	US: California
Micranthes aprica (Greene) Small	RS45	R. L. Stubbs 45 (FLAS)	US: Oregon
Micranthes atrata (Engl.) Losinsk.	RS105	R. L. Stubbs 105 (KUN)	China
Micranthes bryophora (A.Gray) Brouillet & Gornall	RS90	R. L. Stubbs 90 (FLAS)	US: Idaho
Micranthes bryophora (A.Gray) Brouillet & Gornall	RS66	R. L. Stubbs 66 (FLAS)	US: California
Micranthes bryophora (A.Gray) Brouillet & Gornall	RS114	R. L. Stubbs 114 (FLAS)	US: California
Micranthes bryophora (A.Gray) Brouillet & Gornall	L05	M. Mancuso 3848 (SRP)	US: Idaho
Micranthes bryophora (A.Gray) Brouillet & Gornall	L04	M. Mancuso 3834 (SRP)	US: Idaho
Micranthes bryophora (A.Gray) Brouillet & Gornall	L03	M. Mancuso 3832 (SRP)	US: Idaho
Micranthes bryophora (A.Gray) Brouillet & Gornall	L02	M. Mancuso 3831 (SRP)	US: Idaho
Micranthes bryophora (A.Gray) Brouillet & Gornall	L01	M. Mancuso 3830 (SRP)	US: Idaho
Micranthes californica (Greene) Small	RS117	R. L. Stubbs 117 (FLAS)	US: California
Micranthes californica (Greene) Small	RS121	R. L. Stubbs 121 (FLAS)	US: California
Micranthes californica (Greene) Small	RS124	R. L. Stubbs 124 (FLAS)	US: California
Micranthes calycina (Sternb.) Gornall & H.Ohba	RS152	R. L. Stubbs 152 (FLAS)	US: Alaska
Micranthes calycina (Sternb.) Gornall & H.Ohba	RS160	R. L. Stubbs 160 (FLAS)	US: Alaska
Micranthes careyana (A.Gray) Small	RS36	R. L. Stubbs 36 (FLAS)	US: North Carolina

Table S1. Continued

Taxa list	accession number (*Micranthes* only)	Voucher information (Herbarium)	collecting locality
Micranthes caroliniana (A.Gray) Small	RS35	R. L. Stubbs 35 (FLAS)	US: North Carolina
Micranthes clavistaminea (Engl. & Irmsch.) Losinsk.)	RS101	R. L. Stubbs 101 (KUN)	China
Micranthes clusii (Gouan) Fern.Prieto, V.Vázquez, Vallines & Cires	L11	P. Carnicero Campmany 1420 (BCB)	Spain
Micranthes davidii (Franch.) Losinsk.	RS102	R. L. Stubbs 102 (KUN)	China
Micranthes divaricate (Engl. & Irmsch.) Losinsk.	RS109	R. L. Stubbs 109 (KUN)	China
Micranthes eriophora (S.Watson) Small	RS40	R. L. Stubbs 40 (FLAS)	US: Arizona
Micranthes eriophora (S.Watson) Small	RS41	R. L. Stubbs 41 (FLAS)	US: Arizona
Micranthes ferruginea (Graham) Brouillet & Gornall	RS87	R. L. Stubbs 87 (FLAS)	US: Montana
Micranthes ferruginea (Graham) Brouillet & Gornall	RS139	R. L. Stubbs 139 (FLAS)	US: Alaska
Micranthes ferruginea (Graham) Brouillet & Gornall	RS155	R. L. Stubbs 155 (FLAS)	US: Alaska
Micranthes ferruginea (Graham) Brouillet & Gornall	RS167	R. L. Stubbs 167 (FLAS)	US: Washington
Micranthes foliolosa (R.Br.) Gornall	RS148	R. L. Stubbs 148 (FLAS)	US: Alaska
Micranthes foliolosa (R.Br.) Gornall	RS158	R. L. Stubbs 158 (FLAS)	US: Alaska
Micranthes fragosa (Suksdorf ex Small) Small	RS63	R. L. Stubbs 63 (FLAS)	US: California
Micranthes fusca (Maxim.) S.Akiyama & H.Ohba	L12	Barkalov, V. Yu (ALA)	Russia
Micranthes gageana (W.W.Sm.) Gornall & H.Ohba	L13	Miyamoto 94 20109	Nepal
Micranthes gaspensis (Fernald) Small	L14	M. L. Fernald 25817 (A)	Canada
Micranthes gormanii (Suksd.) Brouillet & Gornall	RS78	R. L. Stubbs 78 (FLAS)	US: Idaho

Table S1. Continued

Taxa list	accession number (*Micranthes* only)	Voucher information (Herbarium)	collecting locality
Micranthes hieraciifolia (Waldst. & Kit. ex Willd.) Haw.	RS140	R. L. Stubbs 140 (FLAS)	US: Alaska
Micranthes hieraciifolia (Waldst. & Kit. ex Willd.) Haw.	RS144	R. L. Stubbs 144 (FLAS)	US: Alaska
Micranthes hieraciifolia (Waldst. & Kit. ex Willd.) Haw.	RS159	R. L. Stubbs 159 (FLAS)	US: Alaska
Micranthes hitchcockiana (Elvander) Brouillet & Gornall	RS57	R. L. Stubbs 57 (FLAS)	US: Oregon
Micranthes hitchcockiana (Elvander) Brouillet & Gornall	RS58	R. L. Stubbs 58 (FLAS)	US: Oregon
Micranthes howellii (Greene) Small	RS116	R. L. Stubbs 116 (FLAS)	US: California
Micranthes howellii (Greene) Small	L15	John McRae 3-17-16 (HSU)	US: California
Micranthes idahoensis (Piper) Brouillet & Gornall	RS72	R. L. Stubbs 72 (FLAS)	US: Idaho
Micranthes idahoensis (Piper) Brouillet & Gornall	RS76	R. L. Stubbs 76 (FLAS)	US: Idaho
Micranthes integrifolia (Hook.) Small	RS119	R. L. Stubbs 119 (FLAS)	US: California
Micranthes japonica (H.Boissieu) S.Akiyama & H.Ohba	L16	Yamasaki 5015	Japan
Micranthes laciniata (Nakai & Takeda) S.Akiyama & H.Ohba	RS107	R. L. Stubbs 107 (KUN)	China
Micranthes lyallii (Engl.) Small	RS138	R. L. Stubbs 138 (FLAS)	US: Alaska
Micranthes lyallii (Engl.) Small	RS143	R. L. Stubbs 143 (FLAS)	US: Alaska
Micranthes lyallii (Engl.) Small	RS83	R. L. Stubbs 83 (FLAS)	US: Montana
Micranthes manchuriensis (Engl.) Gornall & H.Ohba	RS108	R. L. Stubbs 108 (KUN)	China
Micranthes marshallii (Greene) Small	RS48	R. L. Stubbs 48 (FLAS)	US: Oregon
Micranthes marshallii (Greene) Small	L26	John McRae 4-29-16 (HSU)	US: California
Micranthes melanocentra (Franch.) Losinsk.	RS104	R. L. Stubbs 104 (KUN)	China

Table S1. Continued

Taxa list	accession number (*Micranthes* only)	Voucher information (Herbarium)	collecting locality
Micranthes melanocentra (Franch.) Losinsk.	RS110	R. L. Stubbs 110 (KUN)	China
Micranthes merkii Elven & D.F.Murray	L07	Kawahara 620 (TI)	Japan
Micranthes merkii Elven & D.F.Murray	L06	C.L. Parker 4527 (ALA)	Russia
Micranthes micranthidifolia (Haw.) Small	RS34	R. L. Stubbs 34 (FLAS)	US: North Carolina
Micranthes micranthidifolia (Haw.) Small	RS37	R. L. Stubbs 37 (FLAS)	US: North Carolina
Micranthes nelsoniana var. aestivalis (Fisch. & C.A.Mey.) Elven & D.F.Murray	RS106	R. L. Stubbs 106 (KUN)	China
Micranthes nelsoniana var. carlottae (Calder & Savile) Gornall & H.Ohba	L17	Karen Dillman 201377 (ALA)	US: Alaska
Micranthes nelsoniana var. cascadensis (Calder & Savile) Gornall & H.Ohba	RS168	R. L. Stubbs 168 (FLAS)	US: Washington
Micranthes nelsoniana var. nelsoniana (D.Don) Small	RS147	R. L. Stubbs 147 (FLAS)	US: Alaska
Micranthes nelsoniana var. nelsoniana (D.Don) Small	RS149	R. L. Stubbs 149 (FLAS)	US: Alaska
Micranthes nelsoniana var. porsildiana (Calder & Savile) Elven & D.F.Murray	RS137	R. L. Stubbs 137 (FLAS)	US: Alaska
Micranthes nidifica (Greene) Small	RS50	R. L. Stubbs 50 (FLAS)	US: Oregon
Micranthes nidifica (Greene) Small	RS54	R. L. Stubbs 54 (FLAS)	US: Oregon
Micranthes nidifica (Greene) Small	RS68	R. L. Stubbs 68 (FLAS)	US: California
Micranthes nivalis (L.) Small	RS146	R. L. Stubbs 146 (FLAS)	US: Alaska
Micranthes nivalis (L.) Small	RS150	R. L. Stubbs 150 (FLAS)	US: Alaska
Micranthes nivalis (L.) Small	L18	Tommy Prestø (TRH)	Norway
Micranthes nudicaulis (D.Don) Gornall & H.Ohba	RS157	R. L. Stubbs 157 (FLAS)	US: Alaska
Micranthes nudicaulis (D.Don) Gornall & H.Ohba	L19	Steffi Ickert-Bond 1964 (ALA)	Russia
Micranthes occidentalis (S.Watson) Small	RS60	R. L. Stubbs 60 (FLAS)	US: Oregon

Table S1. Continued

Taxa list	accession number (*Micranthes* only)	Voucher information (Herbarium)	collecting locality
Micranthes occidentalis (S.Watson) Small	RS55	R. L. Stubbs 55 (FLAS)	US: Oregon
Micranthes occidentalis (S.Watson) Small	RS53	R. L. Stubbs 53 (FLAS)	US: Oregon
Micranthes occidentalis(S.Watson) Small	RS75	R. L. Stubbs 75 (FLAS)	US: Idaho
Micranthes occidentalis (S.Watson) Small	RS95	R. L. Stubbs 95 (FLAS)	US: Montana
Micranthes occidentalis/apetala	RS69	R. L. Stubbs 69 (FLAS)	US: Idaho
Micranthes odontoloma (Piper) A.Heller	RS165	R. L. Stubbs 165 (FLAS)	US: Washington
Micranthes odontoloma (Piper) A.Heller	RS169	R. L. Stubbs 169 (FLAS)	US: Washington
Micranthes odontoloma (Piper) A.Heller	RS82	R. L. Stubbs 82 (FLAS)	US: Montana
Micranthes oregana (Howell) Small	RS77	R. L. Stubbs 77 (FLAS)	US: Idaho
Micranthes oregana (Howell) Small	RS46	R. L. Stubbs 46 (FLAS)	US: Oregon
Micranthes oregana (Howell) Small	RS65	R. L. Stubbs 65 (FLAS)	US: California
Micranthes pallida (Wall. ex Ser.) Losinsk.	RS103	R. L. Stubbs 103 (KUN)	China
Micranthes palmerii Bush	RS125	R. L. Stubbs 125 (FLAS)	US: Arkansas
Micranthes palmerii Bush	RS126	R. L. Stubbs 126 (FLAS)	US: Arkansas
Micranthes palmerii Bush	RS130	R. L. Stubbs 130 (FLAS)	US: Arkansas
Micranthes palmerii Bush	RS131	R. L. Stubbs 131 (FLAS)	US: Arkansas
Micranthes pensylvanica (L.) Haw.	RS100	R. L. Stubbs 100 (FLAS)	US: Missouri
Micranthes petiolaris (Raf.) Bush	RS38	R. L. Stubbs 38 (FLAS)	US: North Carolina
Micranthes pseudopallida (Engl. & Irmsch.) Losinsk.	L20	Chungtien-Lijiand-Dali Expedition CLD-90 no 989	Nepal
Micranthes razshivinii (Zhmylev) Brouillet & Gornall	RS141	R. L. Stubbs 141 (FLAS)	US: Alaska
Micranthes razshivinii (Zhmylev) Brouillet & Gornall	RS142	R. L. Stubbs 142 (FLAS)	US: Alaska
Micranthes razshivinii (Zhmylev) Brouillet & Gornall	RS145	R. L. Stubbs 145 (FLAS)	US: Alaska
Micranthes redofski (Adams) Elven & D.F.Murray	L21	H. Solstad and R. Elven 04/1074 (ALA)	Russia

Table S1. Continued

Taxa list	accession number (*Micranthes* only)	Voucher information (Herbarium)	collecting locality
Micranthes reflexa (Hook.) Small	RS133	R. L. Stubbs 133 (FLAS)	US: Alaska
Micranthes reflexa (Hook.) Small	RS134	R. L. Stubbs 134 (FLAS)	US: Alaska
Micranthes rhomboidea (Greene) Small	RS42	R. L. Stubbs 42 (FLAS)	US: Arizona
Micranthes rhomboidea (Greene) Small	RS79	R. L. Stubbs 79 (FLAS)	US: Idaho
Micranthes rufidula Small	RS162	R. L. Stubbs 162 (FLAS)	US: Alaska
Micranthes rufidula Small	RS52	R. L. Stubbs 52 (FLAS)	US: Oregon
Micranthes rufopilosa (Hult.n) D.F. Murray & Elven	L22	Bruce A. Bennett, Syd Cannings 12-0243 (ALA)	Canada
Micranthes spicata (D.Don) Small	RS153	R. L. Stubbs 153 (FLAS)	US: Alaska
Micranthes spicata (D.Don) Small	RS161	R. L. Stubbs 161 (FLAS)	US: Alaska
Micranthes stellaris (L.) Galasso, Banfi & Soldano	L23	P. Carnicero Campmany 1420 (BCB)	Spain
Micranthes stellaris (L.) Galasso, Banfi & Soldano	L24	Per Arne Pedersen V86729 (ALA)	Norway
Micranthes subapetala (E.E.Nelson) Small	RS89	R. L. Stubbs 89 (FLAS)	US: Montana
Micranthes subapetala (E.E.Nelson) Small	RS97	R. L. Stubbs 97 (FLAS)	US: Montana
Micranthes subapetala (E.E.Nelson) Small	RS98	R. L. Stubbs 98 (FLAS)	US: Montana
Micranthes tempestiva (Elvander & Denton) Brouillet & Gornall	RS91	R. L. Stubbs 91 (FLAS)	US: Montana
Micranthes tempestiva (Elvander & Denton) Brouillet & Gornall	RS81	R. L. Stubbs 81 (FLAS)	US: Montana
Micranthes tempestiva (Elvander & Denton) Brouillet & Gornall	RS85	R. L. Stubbs 85 (FLAS)	US: Montana
Micranthes tenuis (Wahlenb.) Small	RS151	R. L. Stubbs 151 (FLAS)	US: Alaska
Micranthes tenuis (Wahlenb.) Small	RS156	R. L. Stubbs 156 (FLAS)	US: Alaska
Micranthes texana (Buckley) Small	RS127	R. L. Stubbs 127 (FLAS)	US: Arkansas
Micranthes texana (Buckley) Small	RS128	R. L. Stubbs 128 (FLAS)	US: Arkansas
Micranthes tischii (Skelly) Brouillet &	RS163	R. L. Stubbs 163 (FLAS)	US: Washington

Table S1. Continued

Taxa list	accession number (*Micranthes* only)	Voucher information (Herbarium)	collecting locality
Gornall			
Micranthes tischii Brouillet & Gornall	RS164	R. L. Stubbs 164 (FLAS)	US: Washington
Micranthes tolmiei (Torr. & A.Gray) Brouillet & Gornall	RS88	R. L. Stubbs 88 (FLAS)	US: Montana
Micranthes tolmiei (Torr. & A.Gray) Brouillet & Gornall	RS74	R. L. Stubbs 74 (FLAS)	US: Idaho
Micranthes tolmiei (Torr. & A.Gray) Brouillet & Gornall	RS67	R. L. Stubbs 67 (FLAS)	US: California
Micranthes tolmiei (Torr. & A.Gray) Brouillet & Gornall	RS166	R. L. Stubbs 166 (FLAS)	US: Washington
Micranthes tolmiei (Torr. & A.Gray) Brouillet & Gornall	RS115	R. L. Stubbs 115 (FLAS)	US: California
Micranthes tolmiei (Torr. & A.Gray) Brouillet & Gornall	RS113	R. L. Stubbs 113 (FLAS)	US: California
Micranthes tolmiei (Torr. & A.Gray) Brouillet & Gornall	RS112	R. L. Stubbs 112 (FLAS)	US: California
Micranthes tolmiei (Torr. & A.Gray) Brouillet & Gornall	RS111	R. L. Stubbs 111 (FLAS)	US: California
Micranthes tolmiei (Torr. & A.Gray) Brouillet & Gornall	L10	K. Dillman 2013120 (ALA)	US: Alaska
Micranthes tolmiei (Torr. & A.Gray) Brouillet & Gornall	L09	G. Johnson 4 (SRP)	US: Idaho
Micranthes tolmiei (Torr. & A.Gray) Brouillet & Gornall	L08	C. Davidson 12066 (SRP)	US: Idaho
Micranthes unalaschensis (Sternb.) Gornall & H.Ohba	L25	Leah Kenney, Robb S. A. Kaler 15 (ALA)	US: Alaska
Micranthes virginiensis (Michx.) Small	RS129	R. L. Stubbs 129 (FLAS)	US: Arkansas
Micranthes virginiensis (Michx.) Small	RS132	R. L. Stubbs 132 (FLAS)	US: Tennessee
Micranthes virginiensis (Michx.) Small	RS39	R. L. Stubbs 38 (FLAS)	US: Missouri
Altingia siamensis Craib		Gaoligong Shan expedition 1997 18235	China

Table S1. Continued

Taxa list	accession number (*Micranthes* only)	Voucher information (Herbarium)	collecting locality
		(E)	
Astilbe microphylla Knoll		Nikko Botanical Garden	Japan
Boykinia aconitifolia Nutt.		Werth s.n. (WS)	US: Virginia
Cascadia nuttallii (Small) A.M.Johnson		WS 21562	US: Washington
Choristylis rhamnoides Harv.		Burrows 4130 (E)	South Africa
Chrysosplenium album Maxim.		Soltis J2534 (WS)	Japan
Chrysosplenium davidianum Decne. ex Maxim.		Boufford 29122 (E)	China
Chrysosplenium nepalense D.Don		Gaoligong Shan expedition 1997 24226 (E)	China
Chrysosplenium sinicum Maxim.		Gaoligong Shan expedition 1997 1185 (E)	China
Glischrocaryon behrii (Schltdl.) Orchard		Conn 2545 (E)	Australia
Itea oldhamii C. K. Schneider		Noshiro 19506 (TI)	Japan
Itea parviflora Hemsl.		Wang 1230 (TI)	Taiwan
Lithophragma bolanderi A.Gray		Davis 66673 (E)	US: California
Mytilaria laosensis Lecomte		Sykes CH336 (E)	China
Pterostemon mexicanus Schauer		Tenorio 17208 (MEXU)	Mexico
Pterostemon rotundifolius Ramirez		Tenorio 20388 (MEXU)	Mexico
Ribes janczewskii Pojark.		Chase 3597 (K)	Kyrgyzstan
Ribes komarovii Pojark.		Gorovoi 6896	Croatia
Saxifraga globulifera Desf.		RBGE (#19813354); Gardner 1242 (E)	United Kingdom (originally Spain)
Saxifraga mertensiana		R. L. Stubbs 49 (FLAS)	US: California
Saxifraga rotundifolia L.		RBGE (# 19581387)	United Kingdom (originally Spain)
Saxifraga rufescens Balf.f.		RBGE (# 19910661); Chungtien 788 (E)	United Kingdom

Table S1. Continued

Taxa list	accession number (*Micranthes* only)	Voucher information (Herbarium)	collecting locality
			(originally China)
Saxifraga taylorii Calder & Savile		RBGE (# 19811909)	United Kingdom (originally Canada)
