# Supplementary material for: A Phylogenomic Perspective on Evolution and Discordance in the Alpine-Arctic Plant Clade Micranthes (Saxifragaceae)
Source: Front Plant Sci. 2020 Feb 7;10:1773. doi: 10.3389/fpls.2019.01773 (PMC7020907; doi:10.3389/fpls.2019.01773)
Supplement: Supplementary file 2 [file DataSheet_2.pdf]

Table S2 Target capture results.

Name	# Reads	Reads Mapped	On Target	Paralog Warnings
M. apetala RS61	7835226	4316945	0.551	14
M. apetala RS84	4313675	2580031	0.598	3
M. aprica RS43	11477631	5709530	0.497	17
M. aprica RS45	3033376	1782431	0.588	3
M. atrata RS105	12830147	5549104	0.433	20
M. bryophora L01	27623187	18140889	0.657	26
M. bryophora L02	9468124	5914263	0.625	18
M. bryophora L03	4612940	2797159	0.606	14
M. bryophora L04	7910542	3616969	0.457	6
M. bryophora L05	9372855	3658635	0.39	10
M. bryophora RS66	10329688	4138995	0.401	11
M. bryophora RS90	4732562	1760970	0.372	7
M. bryophora RS114	12310707	8306070	0.675	25
M. californica RS117	5348505	3163754	0.592	6
M. californica RS121	4189436	2492032	0.595	7
M. californica RS121	5401586	3274231	0.606	6
M. californica RS124	2072399	1154288	0.557	5
M. calycina RS152	1707547	1000868	0.586	7
M. calycina RS160	13828946	6277138	0.454	9
M. careyana RS36	11671910	5442435	0.466	10
M. caroliniana RS35	10833639	3454568	0.319	10
M. clavistaminea RS101	6129548	4086197	0.667	13
M. clusii L11	6500855	2016735	0.31	0
M. davidii RS102	9107395	2935109	0.322	11
M. divaricata RS109	4769071	2695855	0.565	4
M. eriophora RS40	13306102	7185460	0.54	17

Table S3. Continued

Name	# Reads	Readzs Mapped	OnTarget	Paralog Warnings
M. eriophora RS41	5533244	3696532	0.668	0
M. ferruginea RS87	3834828	2426006	0.633	0
M. ferruginea RS139	6249920	4299577	0.688	0
M. ferruginea RS155	10941242	4925480	0.45	19
M. ferruginea RS167	2948585	1698074	0.576	0
M. foliolosa RS148	3537461	2242917	0.634	0
M. foliolosa RS158	754332	344650	0.457	0
M. fragosa RS63	2103663	673251	0.32	0
M. fusca L12	1691944	1280888	0.757	0
M. gageana L13	17414889	4683378	0.269	12
M. gaspensis L14	3864374	2239896	0.58	8
M. gormanii RS78	4182367	2308900	0.552	11
M. hieracifolia RS140	1895214	1124544	0.593	8
M. hieracifolia RS144	11449178	5854084	0.511	70
M. hieracifolia RS159	4329928	2430831	0.561	3
M. hitchcockiana RS57	3291497	2119085	0.644	6
M. hitchcockiana RS58	2615367	1522100	0.582	7
M. howellii L15	4472897	2577124	0.576	9
M. howellii RS116	3568510	2135285	0.598	2
M. idahoensis RS72	542573	214599	0.396	0
M. idahoensis RS76	12913374	6950593	0.538	15
M. integrifolia RS119	2456004	1387803	0.565	2
M. integrifolia RS119	3671319	1961024	0.534	12
M. japonica L16	6740581	1571356	0.233	10
M. laciniata RS107	2661715	1588362	0.597	5
M. lyallii RS83	13187866	5245942	0.398	52
M. lyallii RS138	9880631	4074454	0.412	16
M. lyallii RS143	2800454	1605602	0.573	0

Table S3. Continued

Name	# Reads	Readzs Mapped	OnTarget	Paralog Warnings
M. manchuriensis RS108	2131075	1084793	0.509	6
M. marshallii L26	10144257	5036315	0.496	8
M. marshallii RS48	15744985	8874171	0.564	8
M. melanocentra RS104	1726732	674314	0.391	0
M. melanocentra RS110	1891155	537053	0.284	0
M. merkii L06	1794466	887335	0.494	0
M. merkii L07	7587931	2668505	0.352	16
M. micranthidifolia RS34	7884558	2788852	0.354	18
M. micranthidifolia RS37	2385218	1466020	0.615	7
M. nelsoniana var. aestivalis RS106	2603221	1360575	0.523	9
M. nelsoniana var. carlottae L17	2317702	1236363	0.533	9
M. nelsoniana var. cascadensis RS168	2707387	1660841	0.613	6
M. nelsoniana var. nelsonian RS147	2477311	1295751	0.523	4
M. nelsoniana var. nelsoniana RS149	3676968	2261447	0.615	4
M. nelsoniana var. porsildiana RS137	4788429	2806674	0.586	4
M. nidifica RS50	4875680	2793889	0.573	8
M. nidifica RS54	7544173	2061543	0.273	9
M. nidifica RS68	4669720	2725910	0.584	10
M. nivalis L18	8023723	4907341	0.612	11
M. nivalis RS146	2596046	1524464	0.587	8
M. nivalis RS146	2346367	1401974	0.598	24
M. nivalis RS150	2946447	1556854	0.528	8
M. nudicaulis L19	2436576	970723	0.398	0
M. nudicaulis RS157	15464317	6151396	0.398	10
M. occidentalis RS53	4134576	2396417	0.58	4
M. occidentalis RS55	3772005	2317898	0.615	4
M. occidentalis RS55	4527468	2812573	0.621	7
M. occidentalis RS60	8554789	2253716	0.263	11

Table S3. Continued

Name	# Reads	Readzs Mapped	OnTarget	Paralog Warnings
M. occidentalis RS75	3275571	1859258	0.568	0
M. occidentalis RS95	3149816	1801404	0.572	7
M. occidentalis\/apetala_RS69	8158228	1333501	0.163	9
M. odontoloma RS82	3596002	2354284	0.655	5
M. odontoloma RS165	1009626	577441	0.572	3
M. odontoloma RS169	8086863	1927164	0.238	32
M. oregana RS46	22030724	10796060	0.49	22
M. oregana RS65	2699541	1616291	0.599	2
M. oregana RS77	4791223	2740683	0.572	0
M. pallida RS103	3279708	1959422	0.597	5
M. palmerii RS125	3818283	2290099	0.6	5
M. palmerii RS126	10153612	3537927	0.348	39
M. palmerii RS130	11158519	5693605	0.51	14
M. palmerii RS131	572228	61630	0.108	0
M. pensylvanica RS100	2636102	1338073	0.508	11
M. petiolaris RS38	3190484	1605361	0.503	10
M. pseudopallida L20	3591511	1789624	0.498	9
M. razshivinii RS141	1906596	1058030	0.555	1
M. razshivinii RS142	3310546	2055852	0.621	0
M. razshivinii RS145	3037456	1735019	0.571	9
M. redofski L21	12039033	6593417	0.548	16
M. reflexa RS133	5796554	3466934	0.598	5
M. reflexa RS134	3615876	2290458	0.633	4
M. rhomboidea RS42	2799136	1693076	0.605	6
M. rhomboidea RS79	14757971	8326026	0.564	13
M. rufidula RS52	3065279	1895870	0.618	2
M. rufidula RS162	1432895	773903	0.54	0
M. rufopilosa H1	1916862	1110853	0.58	6

Table S3. Continued

Name	# Reads	Readzs Mapped	OnTarget	Paralog Warnings
M. spicata RS153	8208315	5641965	0.687	11
M. spicata RS161	487075	174903	0.359	0
M. stellaris L23	1610978	978940	0.608	0
M. stellaris L24	11451451	4919154	0.43	36
M. subapetala RS89	4701563	2961162	0.63	3
M. subapetala RS97	9524296	3705766	0.389	14
M. subapetala RS98	2931781	1741898	0.594	9
M. tempestiva RS81	3599660	2132121	0.592	10
M. tempestiva RS85	4742916	2588434	0.546	0
M. tempestiva RS91	4798397	2583000	0.538	0
M. tenuis RS151	3384691	2163956	0.639	14
M. tenuis RS156	3552100	2317077	0.652	9
M. texana RS127	7043573	2471580	0.351	6
M. texana RS128	8137175	2665951	0.328	4
M. tischii RS163	8672934	2998459	0.346	4
M. tischii RS164	12866523	4412346	0.343	5
M. tolmiei L08	2246549	853273	0.38	0
M. tolmiei L09	4918238	369687	0.075	1
M. tolmiei L10	7027405	570350	0.081	3
M. tolmiei RS67	8568165	1325903	0.155	3
M. tolmiei RS74	6973826	2600949	0.373	3
M. tolmiei RS88	1927445	627224	0.325	0
M. tolmiei RS111	8721785	2645350	0.303	6
M. tolmiei RS112	553517	177577	0.321	0
M. tolmiei RS113	4616943	2879547	0.624	2
M. tolmiei RS115	4817093	3060177	0.635	1
M. tolmiei RS166	4606622	2815669	0.611	6
