# Supplementary material for: A Phylogenomic Perspective on Evolution and Discordance in the Alpine-Arctic Plant Clade Micranthes (Saxifragaceae)
Source: Front Plant Sci. 2020 Feb 7;10:1773. doi: 10.3389/fpls.2019.01773 (PMC7020907; doi:10.3389/fpls.2019.01773)
Supplement: Supplementary file 3 [file DataSheet_3.pdf]

Table S3. Summary statistics for phylogenetic datasets

Dataset	Alignment Length	% Missing	% Variable	% Parsimony Informative
PLASTID	50009	44.2	29.8	20.5
EXON	665502	21.7	67.3	46.5
