# Supplementary material for: A Phylogenomic Perspective on Evolution and Discordance in the Alpine-Arctic Plant Clade Micranthes (Saxifragaceae)
Source: Front Plant Sci. 2020 Feb 7;10:1773. doi: 10.3389/fpls.2019.01773 (PMC7020907; doi:10.3389/fpls.2019.01773)
Supplement: Figure S1 — Heatmap showing success of target capture. Darker colors represent higher capture success. [file Image_1.pdf]

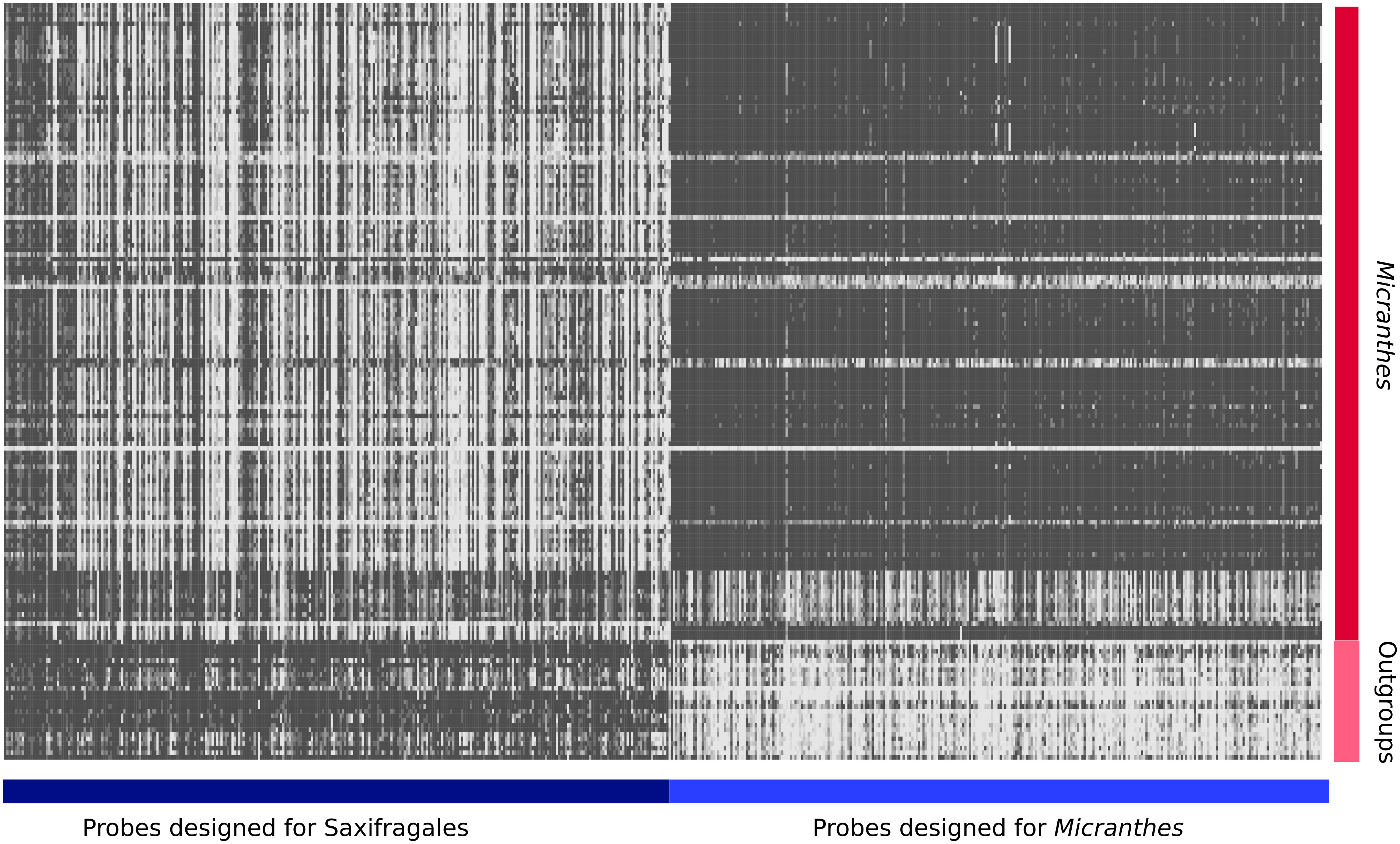

Figure S1. Heatmap showing success of target capture. Darker colors represent higher capture success.
