# Supplementary figures and images for: A Phylogenomic Perspective on Evolution and Discordance in the Alpine-Arctic Plant Clade Micranthes (Saxifragaceae)
Source: Front Plant Sci. 2020 Feb 7;10:1773. doi: 10.3389/fpls.2019.01773 (PMC7020907; doi:10.3389/fpls.2019.01773)

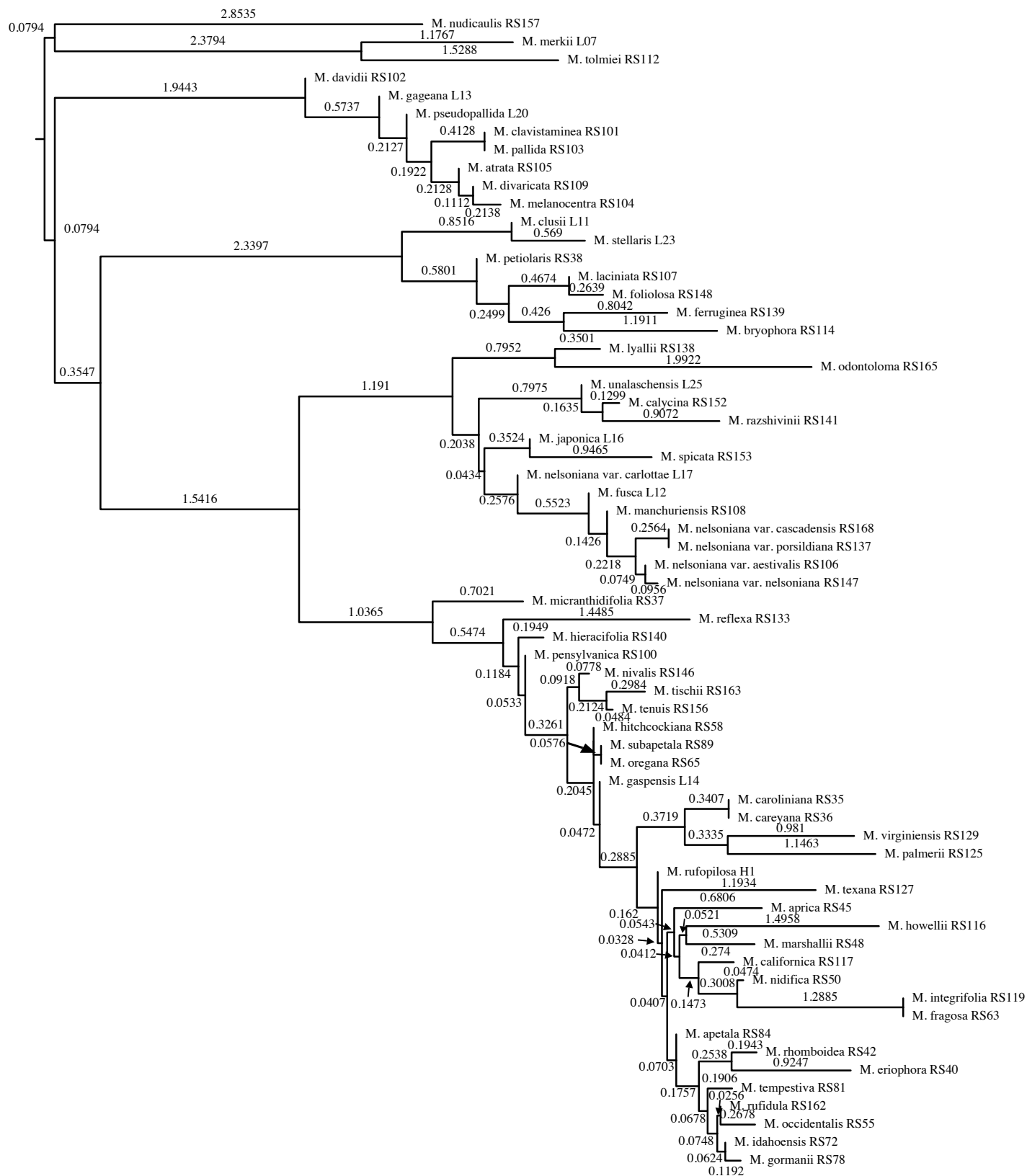

Supplement: Figure S2 — ASTRAL-II topology with single species accessions and outgroups removed. Branch lengths labeled in coalescent units. [file Image_2.pdf]

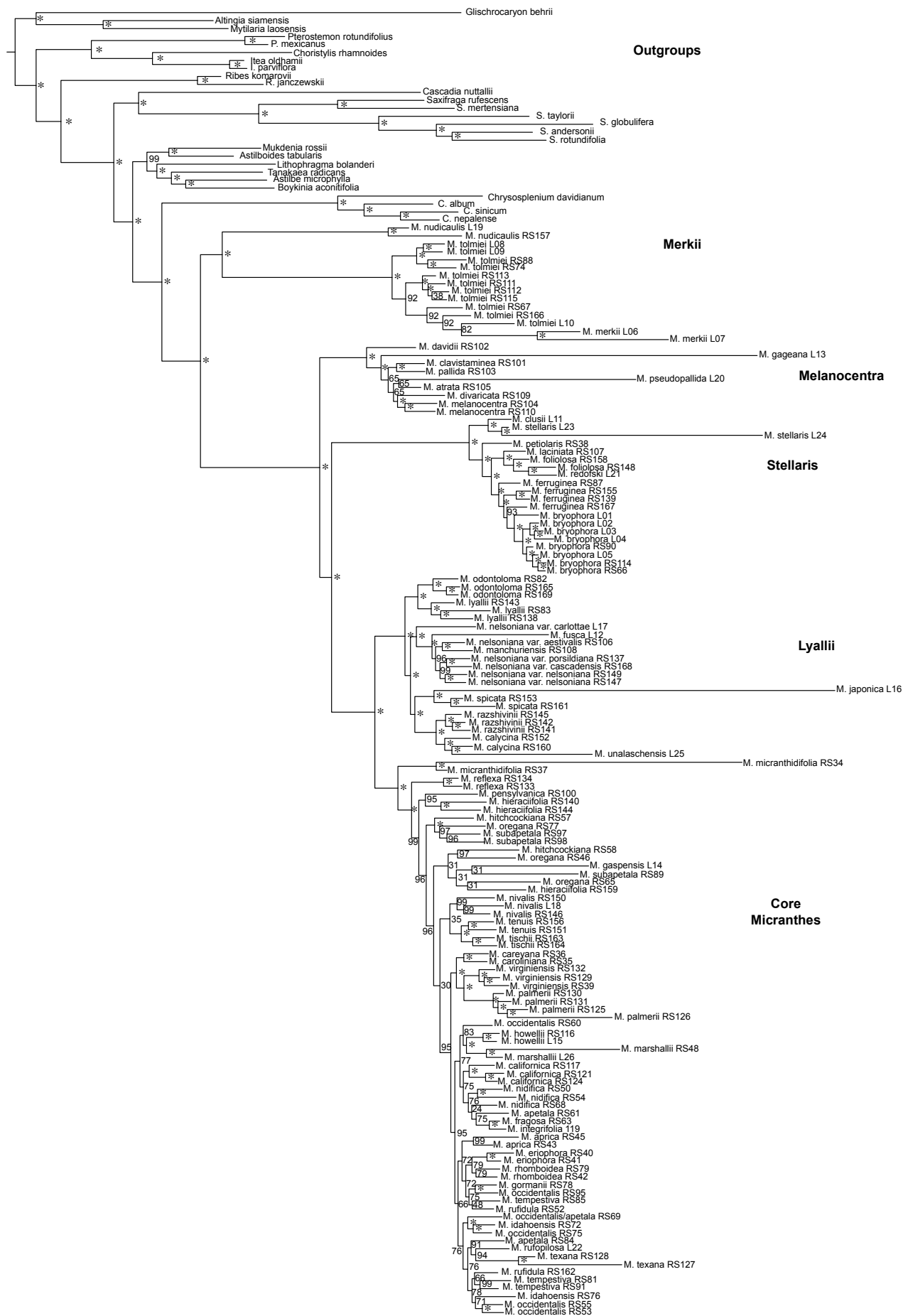

Supplement: Figure S3 — RAxML analysis of concatenated nuclear dataset. Nodes are labeled with bootstrap support values. [file Image_3.pdf]

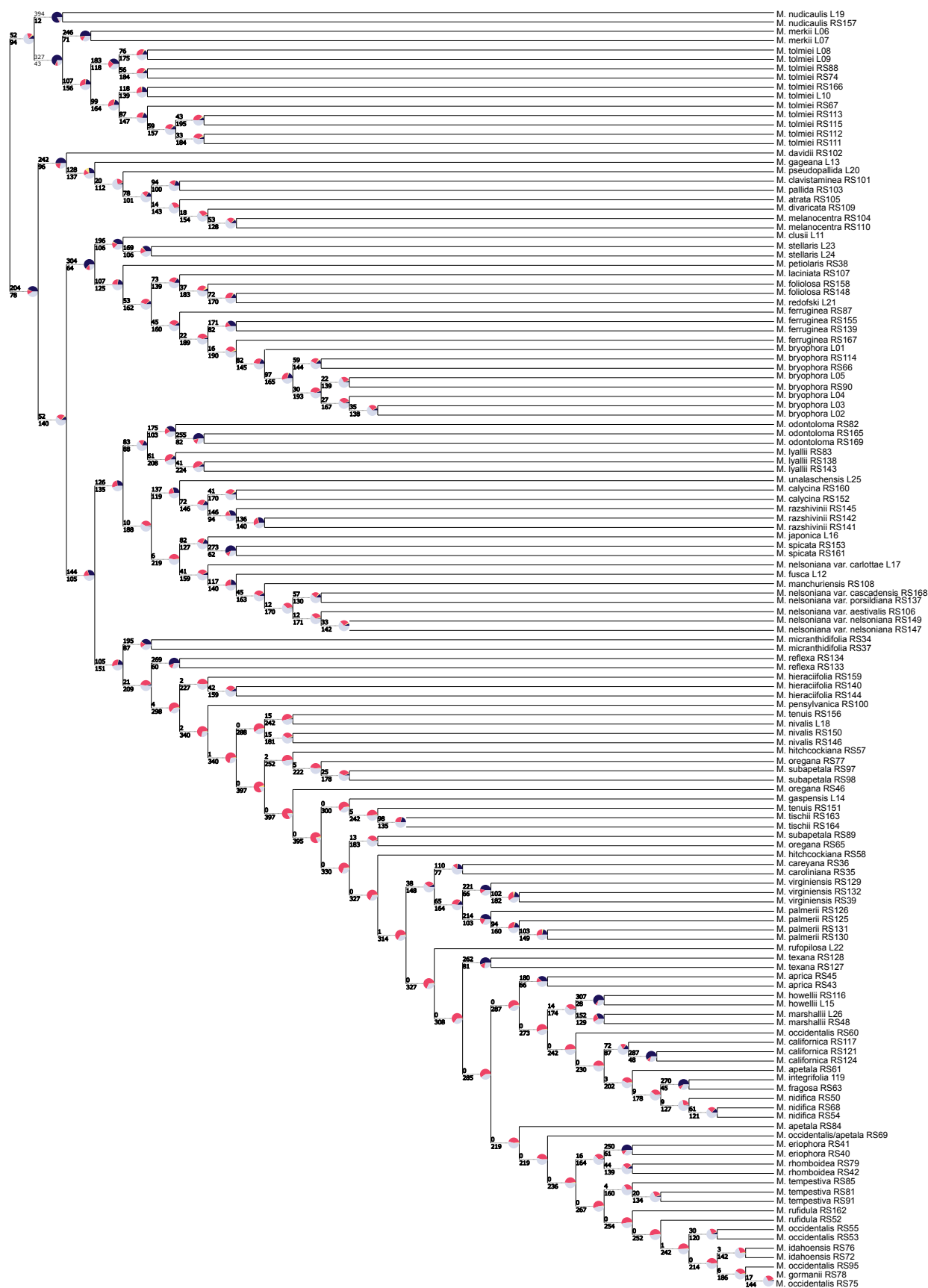

Supplement: Figure S4 — PhyParts results depicting gene tree conflict with the ASTRAL-II species topology. Pie charts show gene tree conflict evaluations at each node as the following: proportion of gene trees in concordance (blue), in conflict (pink), agreeing with the dominant alternative topology (yellow), and unsupported with less than 70% BS (gray). The number above the branch is the number of gene trees that agree with the relationships at that node and the number below the branch is the number of gene trees that are in conflict with that node. Total number of trees is 481. [file Image_4.pdf]

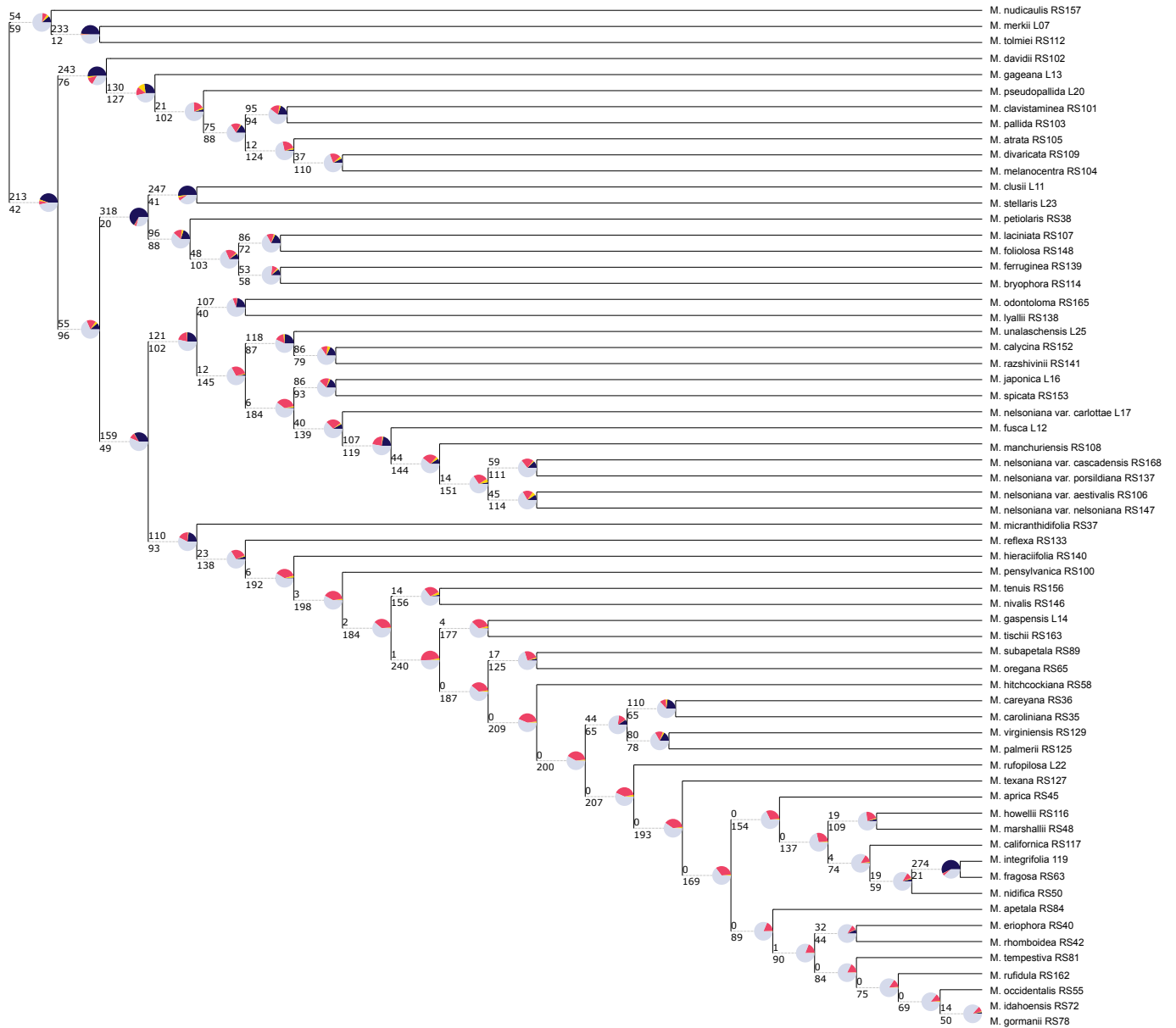

0.1

Supplement: Figure S5 — PhyParts results depicting gene tree conflict with the ASTRAL-II species topology where all but one accession per species were removed in the species tree and gene trees. Pie charts show gene tree conflict evaluations at each node as the following: proportion of gene trees in concordance (blue), in conflict (pink), agreeing with the dominant alternative topology (yellow), and unsupported with less than 70% BS (gray). The number above the branch is the number of gene trees that agree with the relationships at that node and the number below the branch is the number of gene trees that are in conflict with that node. Total number of tree is 478. [file Image_5.pdf]

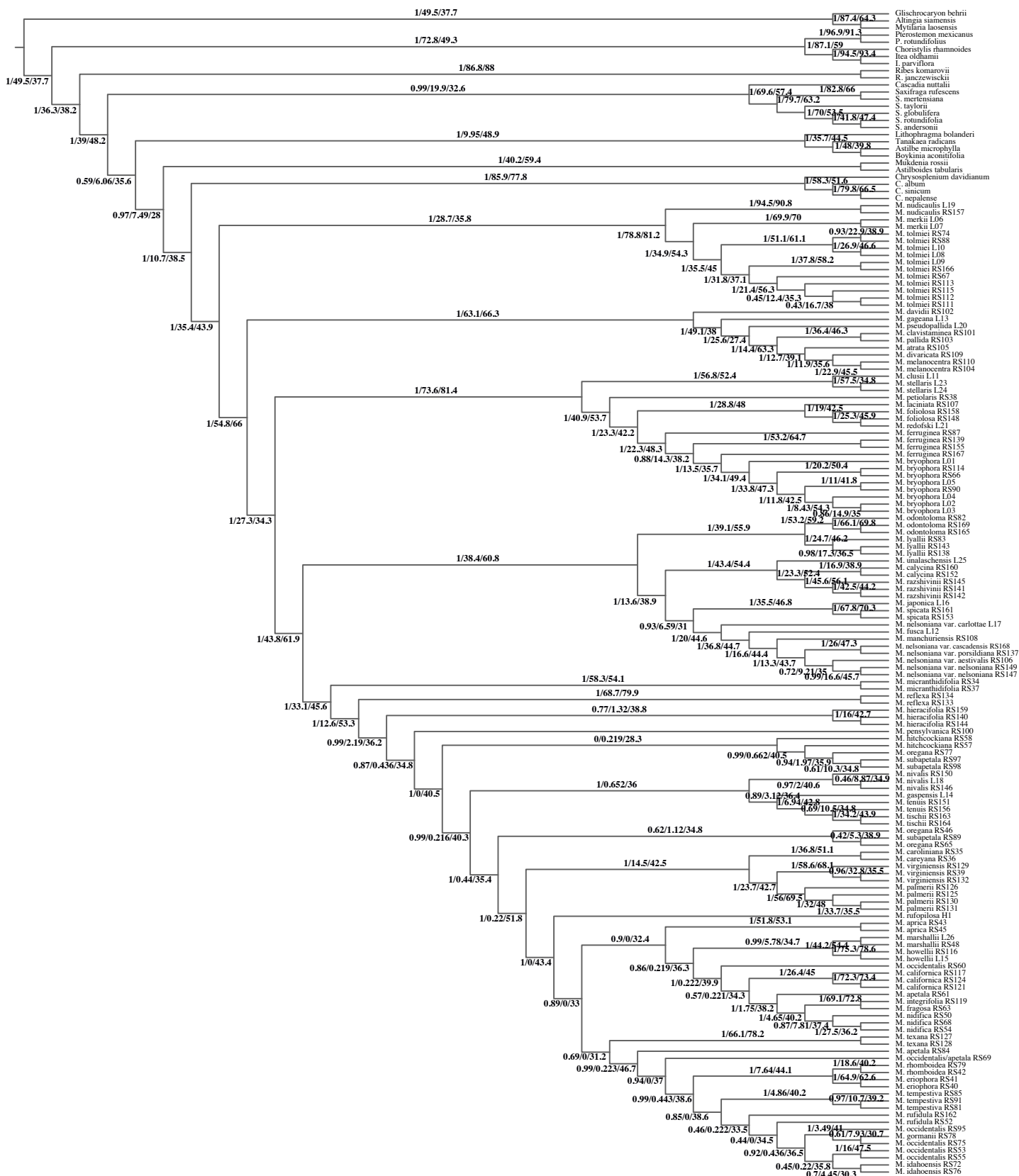

Supplement: Figure S6 — Tree showing posterior probability, gene concordance factor, and sequence concordance faster (in that order) for each branch. The gCF is the percentage of decisive gene trees containing that branch. The sCF is the percentage of alignment sites supporting a branch in the reference tree. Note that unlike gCF values, the sCF values sum to 100% because sCF values are calculated by comparing the three possible resolutions of quartet around a node. [file Image_6.pdf]

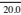

Supplement: Figure S7 — BEAST dating analysis with SortaDate genes. Ages of nodes shown in millions of years and as the 95% HPD. [file Image_7.pdf]

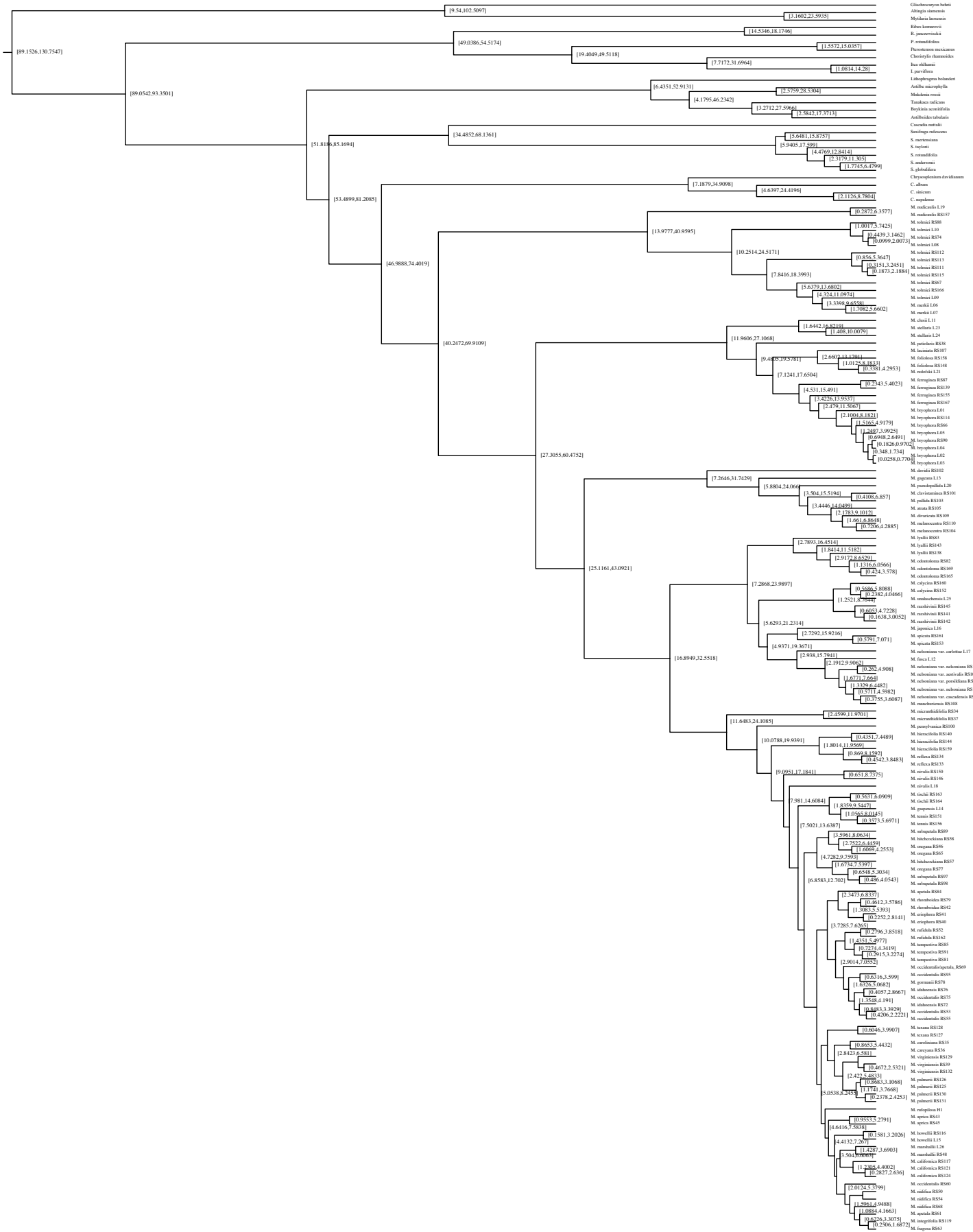

Supplement: Figure S8 — BEAST dating analysis with RF genes. Ages of nodes shown in millions of years and as the 95% HPD. [file Image_8.pdf]
